# Uncovering the Neural Correlates of Anhedonia Subtypes in Major Depressive Disorder: Implications for Intervention Strategies

**DOI:** 10.3390/biomedicines11123138

**Published:** 2023-11-24

**Authors:** Yudan Ding, Yangpan Ou, Haohao Yan, Feng Liu, Huabing Li, Ping Li, Guangrong Xie, Xilong Cui, Wenbin Guo

**Affiliations:** 1 Department of Psychiatry, National Clinical Research Center for Mental Disorders, National Center for Mental Disorders, The Second Xiangya Hospital of Central South University, Changsha 410011, China; dandandding@gmail.com (Y.D.); yanhaohao1995@gmail.com (H.Y.); xiegr2000@126.com (G.X.); 2 Department of Radiology, Tianjin Medical University General Hospital, Tianjin 300052, China; feng_liu6@163.com; 3 Department of Radiology, The Second Xiangya Hospital of Central South University, Changsha 410011, China; huabingli3350@csu.edu.cn; 4 Department of Psychiatry, Qiqihar Medical University, Qiqihar 161006, China; lipingchxyy@163.com

**Keywords:** major depressive disorder, mental health, anhedonia, subgroup, neuroimaging, fMRI, functional connectivity, reward network, default mode network, intervention strategies

## Abstract

Major depressive disorder (MDD) represents a serious public health concern, negatively affecting individuals’ quality of life and making a substantial contribution to the global burden of disease. Anhedonia is a core symptom of MDD and is associated with poor treatment outcomes. Variability in anhedonia components within MDD has been observed, suggesting heterogeneity in psychopathology across subgroups. However, little is known about anhedonia subgroups in MDD and their underlying neural correlates across subgroups. To address this question, we employed a hierarchical cluster analysis based on Temporal Experience of Pleasure Scale subscales in 60 first-episode, drug-naive MDD patients and 32 healthy controls. Then we conducted a connectome-wide association study and whole-brain voxel-wise functional analyses for identified subgroups. There were three main findings: (1) three subgroups with different anhedonia profiles were identified using a data mining approach; (2) several parts of the reward network (especially pallidum and dorsal striatum) were associated with anticipatory and consummatory pleasure; (3) different patterns of within- and between-network connectivity contributed to the disparities of anhedonia profiles across three MDD subgroups. Here, we show that anhedonia in MDD is not uniform and can be categorized into distinct subgroups, and our research contributes to the understanding of neural underpinnings, offering potential treatment directions. This work emphasizes the need for tailored approaches in the complex landscape of MDD. The identification of homogeneous, stable, and neurobiologically valid MDD subtypes could significantly enhance our comprehension and management of this multifaceted condition.

## 1. Introduction

Major depressive disorder (MDD) represents a pervasive mental health concern with substantial prevalence and far-reaching societal implications [1]. Nevertheless, despite its ubiquity, the precise underlying mechanisms and reliable biomarkers of MDD remain elusive. A complex interplay of heterogeneous symptoms, symptom clusters, and evolving diagnostic criteria further obscures the path to substantial progress [2,3,4]. Consequently, there arises a pressing need for the exploration and refinement of valid phenotypes that could enhance our comprehension and management of this debilitating disorder.

Anhedonia, traditionally defined as ‘loss of pleasure’, is a cardinal presentation of MDD. Instead of a unitary construct, accumulating evidence has suggested that anhedonia is a multifaceted concept encompassing a complex of deficits in the reward system, such as motivation, reward learning, anticipation, and consummatory pleasure [5]. Importantly, shared and distinct neurobiological mechanisms may underlie the different facets of anhedonia [6].

To date, considerable efforts have been made to decipher anhedonia in MDD; most of the studies, however, did not separate the different aspects of the reward system or focused heavily on consummatory pleasure [5]. For example, the Snaith–Hamilton Pleasure Scale (SHAPS), the “first generation” validated self-report questionnaire, only measures consummatory pleasure [7]. Compared with healthy controls, a lower level of self-report anticipatory pleasure was reported by patients with MDD, whereas both intact and impaired consummatory pleasures were reported in individuals with depression [8,9,10]. The differences in potential MDD subgroups and the methodology used to measure anhedonia may be crucial contributors to such inconsistency. The variability of anhedonia components in MDD might reflect the heterogeneity of psychopathology in specific subgroups.

Current subtyping of MDD in existing diagnostic criteria is based solely on clinical symptoms. For instance, melancholic depression, characterized by pervasive anhedonia with diurnal variation, psychomotor disturbances, and impaired cognitive functions, is regarded as a specifier of MDD in the Diagnostic and Statistical Manual for Mental Disorders Fifth Edition (DSM-5) [11,12]. Generally, melancholic depression has some features that are distinguishable from non-melancholic ones. It is associated with higher heritability and greater biological perturbations [13]. Melancholic MDD can be quantitatively and qualitatively distinguished from non-melancholic depression and healthy controls by a specific neurocognitive function profile [14]. Moreover, blunted reward processing was observed in an MDD sample with impaired mood reactivity (a core feature of melancholic depression) rather than in all MDD cases [15], and such reward-related impairment is also prominent in remitted melancholic depression [16]. From a therapeutic perspective, antidepressant medication is more likely to be effective than psychotherapy (specifically cognitive behavior therapy) for patients with melancholic depression [17].

However, such subtyping does not accurately reflect the variability in the spectrum of the anhedonia components in MDD, and very few studies have investigated anhedonia subgroups in MDD [18]. Lin et al. conducted an explorative study and identified two anhedonia subgroups using a data-driven approach [18]. Better characterization of anhedonia heterogeneity in MDD can potentially facilitate neurobiological studies to define more valid subtypes of MDD and can also help to develop more effective intervention strategies targeting reward deficits.

Mounting evidence from neuroimaging studies has suggested that the mesolimbic circuit and additional brain areas, such as the lateral hypothalamus and dorsal striatum, are involved in reward processing [19]. Specifically, previous studies suggested that the ventral striatum plays an important role in reward anticipation, and the ventral pallidum and nucleus accumbens (NAc) are the central hubs mediating consummatory pleasure [20]. Additionally, medial frontal areas, such as the medial prefrontal cortex (mPFC) and anterior cingulate cortex (ACC), have potential roles in these processes [20].

The majority of these studies were based on behavioral tasks, such as the monetary incentive delay (MID) task, which was designed to tap into the prediction (or anticipation, associated with anticipatory anhedonia) and experience (or feedback, associated with consummatory anhedonia) phases of reward [21,22]. Resting-state fMRI is increasingly utilized to investigate reward deficits in MDD. These studies underscored the importance of striatal circuitry in the neural basis of anhedonia in MDD using pre-defined regions of interest (ROIs) [23,24,25], though this traditional seed-based approach may increase the possibility for false positives and weaken the potentially important effects of other brain regions.

The treatment of anhedonia in MDD poses a significant challenge, encompassing both primary psychological approaches like cognitive behavioral therapy and pharmacological treatments [26]. Recognizing these unmet needs, recent years have witnessed the development of some targeted psychological interventions. For example, Behavioral Activation Treatment encourages activation by enhancing value-guided behaviors and minimizing avoidance behaviors, aiming to maximize engagement with potential sources of reinforcement [27]. There is promising evidence that the modulation of reward-related neural circuits may underlie the reduction of anhedonia through Behavioral Activation [27]. With respect to pharmacological treatments, kappa opioid receptor (KOR) antagonists had been suggested as a potential treatment for anhedonia [28,29,30]. These findings indicate that patients with varying anhedonia deficits may benefit from personalized therapeutic strategies targeting specific aspects of the reward system.

**Figure 1:**
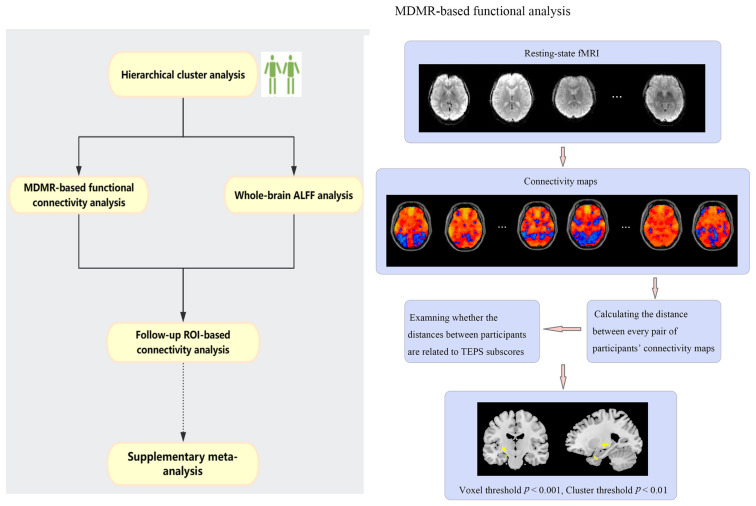
Schematic of the data analysis procedure. Detailed MDMR results are shown in Section 3.

This study’s primary objectives are twofold: to explore anhedonia subgroups within MDD and healthy controls based on scores from the Temporal Experience of Pleasure Scale (TEPS) subscales, namely anticipatory pleasure (TEPS-anti) and consummatory pleasure (TEPS-con). Additionally, we aim to investigate the neural correlates associated with differences in anhedonia across these subgroups. To achieve this, we first performed a connectome-wide association study based on a multivariate distance-based matrix regression (MDMR) approach to identify brain areas which were related to anticipatory and consummatory pleasure. Next, considering our focus on discerning group disparities in the connectivity pattern driving the MDMR outcome, we opted for a whole-brain voxel-wise analysis (using the Amplitude of Low Frequency Fluctuations [ALFF] method) prior to subsequent seed-based analyses. Then, using the cluster(s), which was significant in the former two steps as ROI(s), post hoc seed-based functional connectivity analyses were conducted to compare across subgroups. The data analysis flowchart is shown in Figure 1. Furthermore, we are intrigued by the potential involvement of distinct reward network hubs in both anticipatory and consummatory pleasures, both in resting-state and task-based fMRI contexts. To explore diverse brain activation patterns associated with reward anticipation and feedback in depressed patients compared to healthy controls, we conducted an additional meta-analysis focusing on task-based fMRI studies.

Our study is guided by three hypotheses: (1) We anticipate identifying specific anhedonia profiles within the MDD sample, revealing different subgroups; (2) we expect that anticipatory and consummatory pleasures will be linked to different brain regions within the reward network; and (3) we hypothesize that variations in anhedonia components between these subgroups will manifest as differing resting-state functional connectivity patterns, especially involving key components of the reward network like the ventral striatum.

## 2. Materials and Methods

### 2.1. Participants

This study was approved by the Medical Research Ethics Committee of the Second Xiangya Hospital of Central South University, China. Sixty-four patients with first-episode drug-naive MDD (31 melancholic MDD and 33 non-melancholic MDD) were recruited from outpatients from 4 May 2014 to 30 December 2016, and 32 healthy controls were recruited from the community using printed advertisements. All participants were right-handed Han Chinese aged 18–45 years and with more than 9 years of education, and gave their written informed consents after fully understanding the study procedures. All patients were independently interviewed and diagnosed by two psychiatrists according to the DSM-IV [31]. The patients had to be in their first major depressive episode with a total score of more than 17 in the 17-item Hamilton Rating Scale for Depression (HAM-D) [32] and an illness duration of less than 12 months. The exclusion criteria were as follows: (1) concurrent axis I disorders; (2) history of any neurological disorders or substance abuse; and (3) exposure to psychotropic medications within at least 2 months. Healthy controls should have no first-degree family history of mental illness. The 17-item HAMD was used to assess depressive severity, and the Chinese versions of the TEPS were used to assess anhedonia. The TEPS has two subscales, designed to distinguish the anticipatory and consummatory aspects of reward [33].

### 2.2. Hierarchical Cluster Analysis

Hierarchical clustering [34] was used to investigate the clustering of data (scores of TEPS-anti- and TEPS-con) and identify latent classes that cluster patients who are similar in anhedonia profiles. It is a method of data mining that builds models based on distance connectivity. At different distances, different clusters will form, which can be represented using a dendrogram. Average linkage clustering, the Unweighted Pair Group Method with Arithmetic Mean, was selected as the linkage criterion:

$$\frac{1}{\left|A\right|\cdot \left|B\right|}\sum_{a\in A}\sum_{b\in B}d\left(a,b\right),$$

where *A* and *B* are two sets of observations, and *d* is a distance. This analysis was performed by using the NbClust package in the R environment. The NbClust package provides 30 indices for determining the number of clusters and proposes the best clustering scheme from the different results obtained by varying all combinations of number of clusters, distance measures, and clustering methods.

Using the same method, we added a supplementary clustering analysis for all participants to examine whether patients with MDD had different anhedonia profiles relative to healthy controls.

To verify and assess the consistency of the subgroup classifications, two additional analyses were conducted. First, leave-one-subject-out analyses were performed to assess the reproducibility of the result obtained from the hierarchical clustering analysis. Second, a separate clustering analysis was carried out using the k-means method, with the optimal cluster number solution determined via the elbow test [35]. These analyses were performed using RStudio (version, R-Tools Technology Inc., Richmond Hill, ON, Canada).

### 2.3. MDMR-Based Functional Connectivity Analysis

All MRI acquisition was obtained from a 3.0 T Siemens scanner (Siemens Healthineers, Erlangen, Germany). Resting-state MRI data acquisition and preprocessing are offered in the Supplementary Materials. An MDMR analysis was performed to identify the connectome-wide associations for TEPS-anti and TEPS-con without biases introduced by a priori network selection. As previously described [36,37,38], the analysis occurred in the following steps (Figure 1). First, standard-space voxel-wise time series data were down-sampled to 4 mm isotropic voxels [38]. Second, connectivity maps (the temporal Pearson’s correlations coefficients between a given voxel and all other gray matter voxels) for each participant were calculated. Third, a Pearson’s correlation coefficient *r*, which measures the spatial correlation of maps between subjects, was computed by comparing the correlation coefficients for corresponding voxels in each subject’s connectivity map. The similarity of connectivity maps between subjects was calculated using the distance metric (1 − *r*). This step yielded a matrix of distances (89 × 89) for each voxel. Finally, MDMR was used to test the relationship of inter-subject differences in TEPS-anti or TEPS-con score with inter-subject distances on the connectivity maps. Covariates, including age, sex, years of education, average framewise displacement [39,40], and total HAM-D scores (given the strong association between anhedonia severity and depression severity), were incorporated into this analysis. This step resulted in a voxel-wise pseudo-F statistic map, which demonstrated how TEPS-anti or TEPS-con scores were reflected in functional connectivity at each voxel, with permutation-based significance testing using 5000 permutations. Gaussian random field (GRF) theory with a voxel threshold of *p* < 0.001 and a cluster threshold of *p* < 0.01 were used to correct for multiple comparison.

### 2.4. Whole-Brain ALFF Analysis

An ALFF analysis was conducted by DPARSF (http://rfmri.org/DPARSF accessed on 2 October 2023) in MATLAB (version 2018a, MathWorks, Natick, MA, USA). ALFF is the mean of amplitudes within a specific frequency domain (here, 0.01–0.1 Hz) [41,42] from a fast Fourier transform of a voxel’s time course. An analysis of covariance (ANCOVA) was performed to compare differences across MDD subgroups on voxel-based ALFF maps, followed by post hoc *t*-tests. Age, sex, years of education, and average framewise displacement were used as covariates. The results were GRF-corrected with a voxel threshold of *p* < 0.01 and a cluster threshold of *p* < 0.05.

### 2.5. Follow-Up ROI-Based Connectivity Analysis

Brain cluster(s) obtained from the former two steps, which were not only associated with anticipatory or consummatory pleasure but also significant in the resting-state ALFF analysis, were used as ROI(s). Then, whole-brain maps of the Z-transformed Pearson’s correlation coefficients were calculated between the time courses of the ROI and other voxels across the whole brain. Statistical analyses were the same as 2.5.

### 2.6. Supplementary Meta-Analysis

An Embase, PubMed and Web of Science database search was performed on all human studies between 1 January 2000 and 1 May 2022. Search terms, inclusion and exclusion criteria, data extraction, and analyses were described in the Supplementary Materials. Software SDM-PSI version 6.22 was used to perform voxel-based meta-analyses of reward anticipation and outcome-related activation differences between MDD and healthy controls, which has been used successfully across multiple neuropsychiatric disorders (https://www.sdmproject.com/ accessed on 2 October 2023) [43,44,45].

## 3. Results

### 3.1. Three MDD Subgroups Identified by Hierarchical Clustering

**Figure 2:**
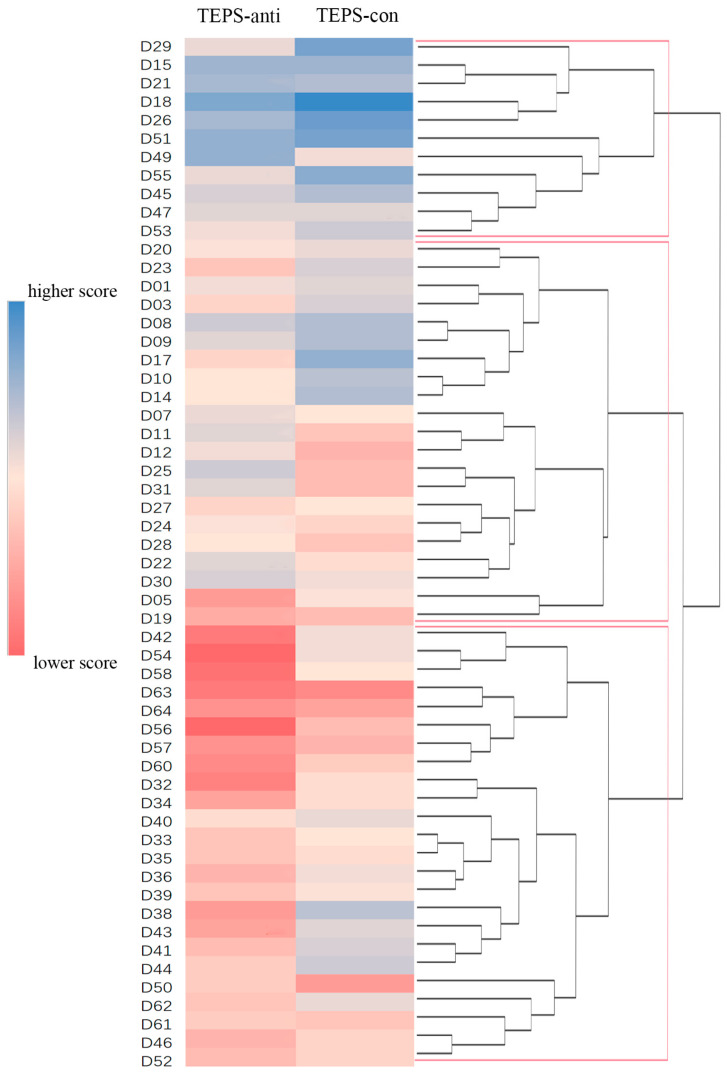
Classification of patients with MDD to 3 clusters based on scores of TEPS-anti and TEPS-con. The dendrogram shows the result of hierarchical clustering, and the heatmap demonstrates scores of TEPS-anti and TEPS-con of each individual. The left column indicated the ID of each individual. Abbreviations: TEPS, the Temporal Experience of Pleasure Scale (TEPS-anti, anticipatory subscale of TEPS; TEPS-con, consummatory subscale of TEPS).

**Figure 3:**
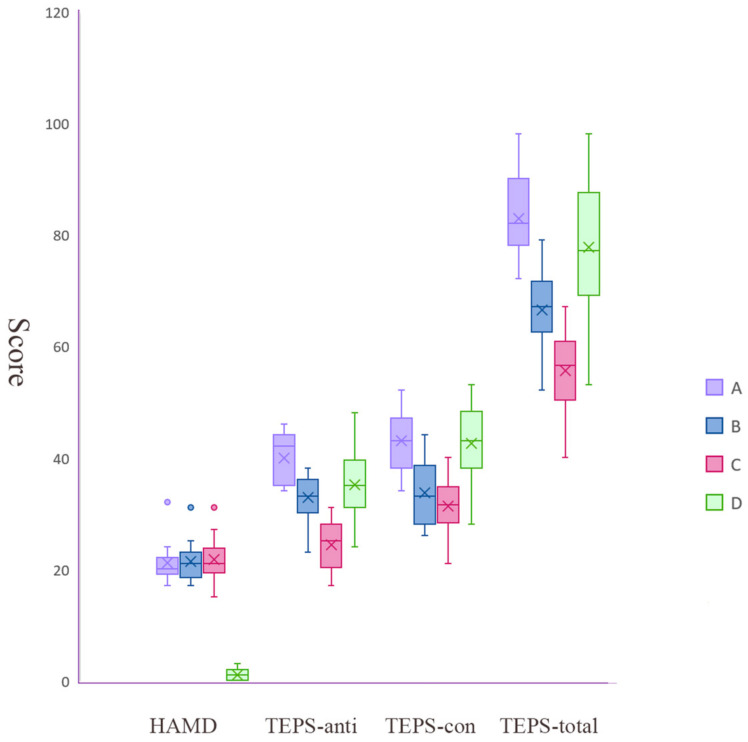
Box graph of scores of TEPS and HAMD of 3 MDD subgroups (A, B, and C) and healthy controls. Group D is the group of healthy controls. Abbreviations: HAMD, Hamilton Rating Scale for Depression; TEPS, the Temporal Experience of Pleasure Scale (TEPS-anti, anticipatory subscale of TEPS; TEPS-con, consummatory subscale of TEPS).

The TEPS scores of two patients were missing and those of two patients were identified as outliers and were excluded. According to hierarchical cluster analysis, the optimal number of categories was three (Figure 2). As shown in Table 1 and Figure 3, one cluster (subgroup A) included 11 patients who had the highest average TEPS scores (preserved ability to experience anticipatory and consummatory pleasure), one cluster (subgroup C) included 26 patients who had the lowest average TEPS scores (the most impaired ability to experience anticipatory and consummatory pleasure), and the other cluster (subgroup B) included 21 patients who had similar average TEPS-anti scores with healthy controls but lower TEPS-con scores in comparison to subgroup A and healthy controls. No significant differences were observed in age, sex, illness duration, and HAMD scores across three subgroups.

The results of the supplementary analysis for all participants indicated that most patients with MD had different anhedonia profiles relative to healthy controls and could be distinguished using hierarchical clustering based on TEPS scores (Figure S2, Supplementary Materials).

Cross validation analysis revealed that 86% of patient combinations supported that the optimal number of clusters was three. A K-means clustering analysis showed that the optimal number of categories suited for all patients was k = 2 (Figure S4A). Cluster 1, including 18 patients, showed higher TEPS scores. Cluster 2, including 38 patients, was characterized by relatively low TEPS scores (Figure S4B). When we compared the results of hierarchical clustering with those of k-means clustering, we found that all patients in hierarchical clustering group C belonged to Cluster 2, while patients in hierarchical clustering group A were exclusively categorized into Cluster 1. Similarly, a k-means clustering analysis exhibited that the optimal number of categories suited for all participants was k = 2 (more details see Supplementary Materials, Figure S5). The substantial overlap between the hierarchical clustering and k-means results provides a degree of verification for the rationale of subtyping MDD into at least two groups.

### 3.2. Resting-State Connectivity to Several Regions within the Reward Network Is Correlated to Anticipatory and Consummatory Pleasure

**Figure 4:**
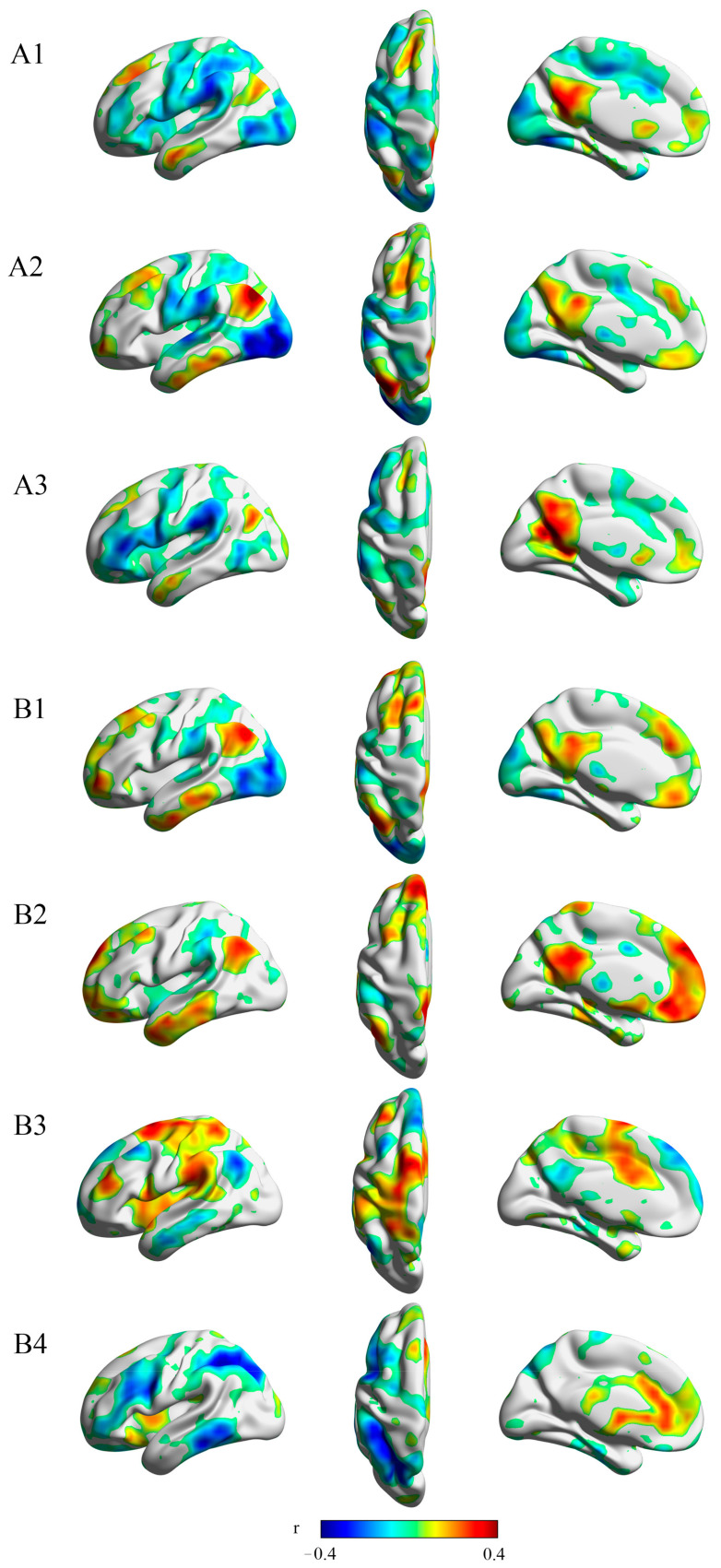
Connectivity patterns that drive MDMR results. The multivariate results of the connectome-wide association study identified the rostral hippocampus (**A1**), ventral pallidum (**A2**), and caudal hippocampus (**A3**), where the overall pattern of connectivity is related to anticipatory pleasure; and the ventral pallidum (**B1**), parahippocampal gyrus (**B2**), caudal hippocampus (**B3**), and superior ACC (**B4**), where the overall pattern of connectivity is related to consummatory pleasure.

According to the MDMR analysis, three regions had a resting-state functional connectivity related to anticipatory pleasure as measured by the TEPS-anti subscale: the rostral hippocampus (z = 3.540), caudal hippocampus (z = 3.719), and ventral pallidum (z = 3.719). Four regions had a functional connectivity correlated to TEPS-con score: the ventral pallidum (z = 3.719), parahippocampal gyrus (z = 3.719), caudal hippocampus (z = 3.432), and superior ACC (z = 3.432). Figure 4 visualizes the patterns of connectivity that drive MDMR results.

### 3.3. ALFF Values Differences across 4 Groups (3 MDD Subgroups and HC)

ANCOVA showed significant differences between groups in the right parahippocampal gyrus (peak MNI = 42, −45, −9), left parahippocampal gyrus (peak MNI = −33, −39, −6), left hippocampus (peak MNI = −30, −18, −15), and right caudate (peak MNI = 18, 21, 6; Table S4, Supplementary Materials). Post hoc analyses showed that subgroup A (highest TEPS scores) and B (in tact TEPS-anti, low TEPS-con) had different ALFF values in the left parahippocampal gyrus/hippocampus (t = −3.11) and left hippocampus/putamen (t = −4.14); subgroup A (highest TEPS scores) and C (lowest TEPS scores) had different ALFF values in the left hippocampus (t = −3.26) and left parahippocampal gyrus/hippocampus (t = −3.15); subgroup B (in tact TEPS-anti, low TEPS-con) and C (lowest TEPS scores) had different ALFF values in the right parahippocampal gyrus (t = 2.98). Additionally, subgroup B (intact TEPS-anti, low TEPS-con) showed an increased ALFF in the right parahippocampal gyrus/hippocampus (t = 3.81) and subgroup C (lowest TEPS scores) showed an increased ALFF in the right caudate (t = 3.35) when compared with healthy controls. No significant results were observed between subgroup A (highest TEPS scores) and healthy controls (based on the results of the former steps, other regions which were not associated with anticipatory/consummatory pleasure were not reported here).

### 3.4. Different Anhedonia Profiles across 3 MDD Subgroups Were Associated with Different Patterns of Within- and Between-Network Connectivity

**Figure 5:**
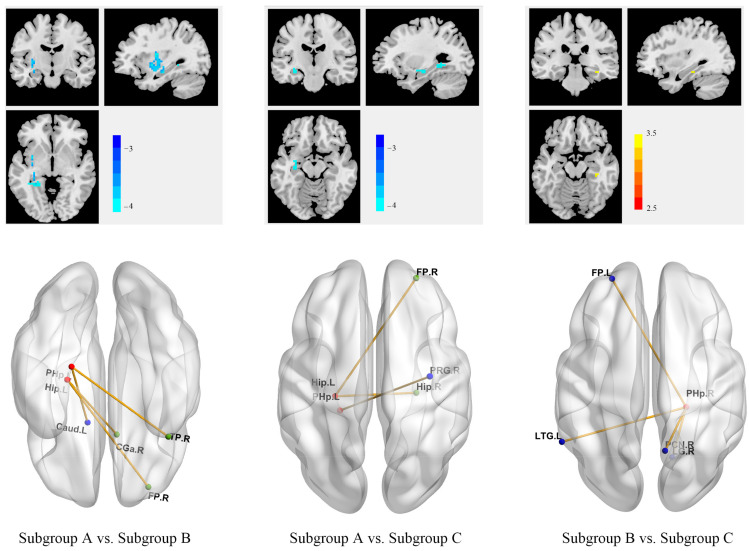
Different anhedonia profiles across 3 MDD subgroups were driven by different patterns of within- and between-network connectivity. ROIs are shown in red. Brain regions with increased connectivity with each ROI are shown in green, and regions with decreased connectivity with each ROI are shown in blue. Abbreviations: Caud, caudate; CGa, anterior cingulate cortex; FP, frontal pole; Hip, hippocampus; LG, lingual gyrus; LTG, lateral temporal gyrus; PHp, parahippocampal gyrus; PCN, precuneous cortex; PRG, precentral gyrus.

The ROI-based functional connectivity analysis was conducted using the regions returned by the former step. As shown in Figure 5, our results demonstrated that connectivity patterns within the reward network and between ROIs and the default mode network were significantly different across subgroups. Relative to subgroups B (in tact TEPS-anti, low TEPS-con) and C (lowest TEPS scores), subgroup A (highest TEPS scores) showed increased functional connectivity between ROIs (hippocampus and parahippocampal gyrus) and the default mode network (DMN) and the frontoparietal network (FPN). Compared with subgroup B (in tact TEPS-anti, low TEPS-con), subgroup C (lowest TEPS scores) had decreased connectivity between ROI (parahippocampal gyrus) and other areas of the reward network and between ROI and DMN/FPN.

### 3.5. MDD Patients Revealed Reduced Activity in Dorsal Striatum during Reward Feedback

As shown in Figure S3 and Table S3 (Supplementary Materials), compared with healthy controls, patients with MDD exhibited increased activation in the supplementary motor area and decreased activation in the cerebellum lobule VI/fusiform gyrus (*p* < 0.01, uncorrected) during reward anticipation. During reward feedback, a less blood-oxygen-level-dependent response in the putamen, caudate, and parahippocampal gyrus was observed in patients with MDD (*p* < 0.001, uncorrected). For more details, see Supplementary Materials.

In summary, there were three main findings: First, three subgroups with different anhedonia profiles were identified using a data mining approach. Second, we found that several parts of the reward network (especially pallidum and dorsal striatum) were associated with anticipatory and consummatory pleasure by combining a functional connectome-wide analysis for our resting-state fMRI sample and meta-analyses for reward task-based fMRI studies. Furthermore, centered on these key hubs of the reward network, different patterns of within- and between-network connectivity contributed to the disparities of anhedonia profiles across three MDD subgroups.

## 4. Discussion

The aim of this study was to explore anhedonia subgroups in a sample of patients with MDD and investigate the underlying neural correlates responsible for these differences across subgroups. We have successfully identified distinct subgroups of patients with MDD based on their anhedonia profiles. This finding is essential as it highlights the heterogeneity within MDD, which may influence treatment strategies and outcomes. Additionally, our investigation of resting-state functional connectivity patterns and neural activity during reward processing has provided valuable insights into the neurobiological underpinnings of anhedonia. These results not only advance our understanding of MDD but also offer potential avenues for developing more targeted interventions. The nuanced relationships between anhedonia profiles and connectivity patterns within the reward network and the observed alterations in dorsal striatum activity during reward feedback provide a foundation for further research into MDD and related therapeutic approaches.

As hypothesized, there were subgroups with different anhedonia profiles in the MDD sample, and a small number of patients with MDD who had preserved ability to experience anticipatory and consummatory pleasure were identified. This result was consistent with previous studies. Rice et al. reported that the symptom of anhedonia was present in about 88.1% of adult patients with MDD, and this rate was much lower in adolescent patients [46]. In another study, anhedonia was not reported in 27% of patients who met criteria for current unipolar major depressive episode [47]. A majority of patients had different levels of deficits in hedonic function: a subgroup demonstrated the most impaired ability to experience anticipatory and consummatory pleasure, and another subgroup had a similar ability to experience anticipatory pleasure to that of healthy controls but impaired ability to experience consummatory pleasure. Previously, Wu et al. observed impaired anticipatory and consummatory pleasures for daily activities in patients with MDD by repeatedly sampling of experiences in daily life [48], and Liu et al. reported lower TEPS-anti and TEPS-con scores in MDD compared with healthy controls [49]. Our findings indicated that anhedonia manifestation within MDD has a meaningful heterogeneity and suggested that results from previous studies may be mainly driven by a subgroup of MDD.

Interestingly, a recent study used latent profile analysis to identify anhedonia in MDD patients based on the Dimensional Anhedonia Rating Scale (DARS) and found that two classes were optimal and a DARS total score of 28.5 was the cut-off value for anhedonia [18]. The cut-off value still had a higher performance in predicting anhedonia in a replication sample. Their results showed that approximately half of the patients were in the non-anhedonia group. The advantage of their study was that they included a large sample of MDD patients (*n* = 533) and a replication sample (*n* = 112). Disparities of the clustering method might also result in the different subgroups identified in our study and their study. Cluster analysis and latent profile analysis are both regarded as “person-oriented analyses”, which use patterns scores across cases to identify individuals who can be grouped together [50]. The two analyses make different assumptions about the data. The former assumes that the cases with the most similar scores across the analysis variables belong in the same cluster, and case membership in clusters is determined. The latter, on the other hand, assumes that latent clusters exist and explains patterns of observed scores across cases. Latent profile analysis assigns cases to classes based on their probability of being in classes, so proper class assignment is not guaranteed and the exact number or percentage of sample members within each class cannot be determined [50]. In addition, their study did not explore the biological underpinnings of anhedonia differences between clusters.

Another important finding of this study was that the consummatory anhedonia was associated with reduced brain activation in the pallidum and dorsal striatum (as indicated by resting-state fMRI analysis and meta-analysis for task-based fMRI studies)—the pivotal hubs of the reward network—in depressed individuals. This result supported previous findings and further indicated that different pallidum and striatum connectivity patterns are involved in consummatory pleasure in MDD subgroups. A transdiagnostic meta-analysis based on ALE has emphasized the importance of the ventral basal ganglia (such as ventral pallidum, caudate, and putamen) in consummatory anhedonia [51]. The ventral pallidum is one of the consistently identified critical brain regions involved in reward processing. It bidirectionally connects with NAc and receives opioid and GABA-ergic signals from NAc [52,53]. The posterior part of the ventral pallidum has an opioid hotspot, which mediates consummatory pleasure [54]. The microinjection of DAMGO—a mu opioid agonist—to the hotspot was sufficient to increase the “liking” reactions of rats to sucrose and the “wanting” for food [54]. Consistent with preclinical evidence, patients with ventral pallidum lesion showed damaged reward responsiveness [55,56].

Unexpectedly, we did not find any significant results in the NAc for anticipatory or consummatory pleasure, which was inconsistent with some previous studies. Using the same connectome-wide method, Sharma et al. found the centrality of the NAc in the pathophysiology of reward deficits across clinical diagnostic categories [37]. However, the focus of their study was reward responsivity measured by the reward sensitivity subscale of the Behavioral Activation Scale (detects personality traits of behavioral approach), whereas we centered on the anticipatory and consummatory aspects of reward and used TEPS as the assessment tool. The measurement disparities between studies may account for the inconsistency. As for the altered activation in the motor and visual cortex during reward anticipation demonstrated by the meta-analysis for task-based fMRI studies, we suggested that it was associated with body movement and visual stimuli during task implementation.

In the present study, we found that patients with an increased level of anhedonia showed reduced functional connectivity between the reward network and DMN. This is consistent with previous evidence which indicated that abnormal functional alterations of the DMN might be one of the features of anhedonia. Decreased functional connectivity between regions within DMN and between DMN and the reward network were reported in several mood and psychotic disorders, including MDD, bipolar disorder, schizophrenia, and patients with posttraumatic anhedonia symptoms [37,57,58]. Based on these results, the dysconnectivity between the reward network and DMN may be associated with transdiagnostic reward deficits.

The association of the dopaminergic system with reward processing has been well-established in numerous preclinical and clinical studies. More recent research has expanded our understanding of reward mediation on a transmitter level, revealing interactions with other neurotransmitter systems like serotonergic, opioid, and glutamatergic systems [59]. This broader perspective suggests that distinct neurotransmitter systems may mediate various aspects of reward, with anticipatory pleasure being predominantly associated with dopaminergic mechanisms, while consummatory pleasure primarily linking to the opioid and serotonergic systems [59]. A comprehensive discussion of this can be found in the Supplementary Materials. Based on a comprehensive review of existing evidence [60,61,62,63,64,65,66,67,68], our hypothesis suggests that neurotransmitter systems, including serotonergic, opioid, glutamatergic, and dopaminergic systems, exhibit varying degrees of disruption within anhedonia subgroups of MDD. Patients exhibiting the most profound impairments in experiencing both anticipatory and consummatory pleasure are expected to demonstrate more severe dysfunction in neurotransmitter systems.

The present findings carry potential implications for personalized medicine in the diagnosis and treatment of MDD. Grouping patients based on their anhedonia profiles could help address the heterogeneity within MDD and guide treatment decisions. For those individuals with the most pronounced impairments in both anticipatory and consummatory pleasure, opioid-modulating drugs or glutamatergic drugs may be considered. Promising research on KOR antagonists has been mentioned in the Introduction, and the glutamatergic drug ketamine has shown favorable effects on anhedonia [69]. In the realm of non-pharmacological interventions, transcranial magnetic stimulation (TMS) is a relevant option. Evidence suggests that anhedonia severity can influence the responsiveness to repetitive TMS, particularly when targeting the dorsomedial prefrontal cortex (DMPFC) [70]. Patients with low anhedonia have shown positive responses to this stimulation, while those with high anhedonia have remained unresponsive. Therefore, individuals with MDD who have the most impaired ability to experience anticipatory and consummatory pleasure might not benefit from rTMS to the DMPFC. In contrast, dorsolateral stimulation appears to have no direct influence on the reward system [71]. Furthermore, deep brain stimulation (DBS) emerges as a promising anti-anhedonic treatment. Several studies have reported its antidepressant and anti-anhedonic effects, particularly when targeting the nucleus accumbens (NAc), in patients with treatment-resistant depression [72,73].

Certain limitations should be mentioned in this study. First, our sample size was relatively small, which could potentially affect the generalization of our findings. Consequently, the current results should be considered as preliminary and tentative. Second, the MDMR method addresses certain challenges posed by mass univariate approaches. However, when applied in a whole-brain analysis with moderate sample sizes, it might still have limitations in detecting the most robust connectivity-phenotype relationships. Nevertheless, it is noteworthy that previous studies have consistently shown that the most substantial connectivity-phenotype relationships across the entire brain are primarily influenced by variations in network topography [74]. Third, this was a cross-sectional study, and the stability and reliability of MDD subgroups needs to be investigated in longitudinal studies. Fourth, the 17-item version of the HAMD excludes various symptoms in the HAMD-21 or, especially, the HAMD-25, which have been used to define atypical depression [75]. The reported prevalence of atypical depression varies between 18–43% [76]. Anhedonia in such patients is highly variable and is subject to external stimuli, making them more challenging to study using the current method. Finally, it is noteworthy that this study investigated potential MDD subtypes based on clinical scales. These scales heavily depend on retrospective recall, which could potentially introduce memory bias among patients and consequently affect the accuracy of measurement results [77]. Given that memory deficits toward mood-accorded negative content are strongly associated with depression [78], we added HAMD scores as a covariate in the analysis, which partly reduced the impact. Nevertheless, exploring biological subtypes can yield a more pronounced impact on further progress. Some studies have sought to parse neuroimaging-based neurophysiological subgroups, using structural and functional MRI, in disorders like schizophrenia [79], bipolar disorder [80], and autism spectrum disorder [81]. The results of our study might provide some insights to guide further research on anhedonia-related, neuroimaging-based MDD subtypes.

## 5. Conclusions

In summary, the present study was the first to investigate anhedonia subgroups in MDD based on the levels of two anhedonia components (anticipatory and consummatory pleasure) and explore neural bases of different anhedonia profiles across MDD subgroups using resting-state fMRI. The ventral pallidum and dorsal striatum had a remarkable relationship with consummatory anhedonia. Different dysconnectivity patterns of these hubs are implicated in different anhedonia features across MDD subgroups. Our findings indicated that interventions related to the opioid system (e.g., opioid agonists) may be potential strategies that treat reward-related deficits in MDD, and suggest that the identification of homogeneous, stable, and neurobiologically valid subtypes of MDD could facilitate better understanding and management of this disorder. Further studies with large sample sizes and longitudinal designs need to validate our findings and may also allow for the evaluation of early interventions that enhance resilience against anhedonia-related psychopathology in specific MDD subgroups.

## Figures and Tables

**Table 1:** Demographic and clinical characteristics of MDD subgroups and healthy controls.

	Subgroup A	Subgroup B	Subgroup C	HC	*p*
N (female %)	11 (54.5%)	21 (66.7%)	24 (79%)	32 (53%)	0.145
	Mean (SD)	Mean (SD)	Mean (SD)	Mean (SD)	
Age	30.27 (7.02)	32.81 (8.04)	27.92 (5.22)	29.59 (5.00)	0.071
Education (years)	14.45 (3.50)	12.38 (3.04)	14.71 (3.07)	14.59 (2.82)	0.039, B < A = C = HC
Duration of illness	5.82 (4.14)	6.67 (4.77)	6.10 (4.27)	-	0.854
HAMD	21.09 (4.06)	21.29 (3.39)	21.67 (3.58)	0.94 (0.95)	<0.001, A = B = C > HC
TEPS-anti	39.82 (4.42)	32.76 (4.04)	24.25 (4.33)	35.13 (6.10)	<0.001, A > B = HC > C
TEPS-con	42.91 (5.52)	33.67 (5.57)	31.29 (4.77)	42.53 (6.63)	<0.001, HC = A > B = C
TEPS-total	82.73 (8.05)	66.43 (6.68)	55.54 (7.14)	77.66 (11.60)	<0.001, HC = A > B > C

Abbreviations: HAMD: Hamilton Rating Scale for Depression; HC, healthy controls; TEPS, the Temporal Experience of Pleasure Scale (TEPS-anti, anticipatory subscale of TEPS; TEPS-con, consummatory subscale of TEPS).

## Data Availability

The data that support the findings of this study are available from the corresponding author, upon reasonable request.

## Supplementary Materials

The following supporting information can be downloaded at: https://www.mdpi.com/article/10.3390/biomedicines11123138/s1. Figure S1: A flow diagram of study selection; Figure S2: Classification of all participants to 2 clusters (the upper: group M, the lower: group N) based on scores of TEPS-anti and TEPS-con. The dendrogram showed the result of hierarchical clustering, and the heatmap demonstrated scores of TEPS-anti and TEPS-con of each individual. The left column indicated the ID of each individual (D is patient and H is healthy control); Figure S3: Differences of brain activation patterns in reward anticipation and feedback between patients with MDD and healthy controls; Figure S4: Elbow test for k-means analysis (A) and k-means clustering results (B) for all patients; Figure S5: Elbow test for k-means analysis (A) and k-means clustering results (B) for all participants; Table S1: Demographic information of all included task-based fMRI studies [82,83,84,85,86,87,88,89,90,91,92,93,94,95,96,97,98,99,100,101,102,103,104,105,106,107,108,109,110,111,112,113,114,115,116,117,118,119,120,121,122,123,124,125,126,127]; Table S2: Summary of methods and results of all included studies; Table S3: Results of meta-analysis for comparative studies with whole-brain method (depressed subjects vs. healthy controls); Table S4: Significant ALFF results (anticipatory and consummatory pleasure-related) across groups (GRF corrected, voxel level < 0.01, cluster level < 0.05).
