# Supplementary material for: Uncovering the Neural Correlates of Anhedonia Subtypes in Major Depressive Disorder: Implications for Intervention Strategies
Source: Biomedicines. 2023 Nov 24;11(12):3138. doi: 10.3390/biomedicines11123138 (PMC10740577; doi:10.3390/biomedicines11123138)
Supplement: Supplementary file 1 [file biomedicines-11-03138-s001.zip › biomedicines-2646002-supplementary.pdf]

Distinct anhedonia components and related subgroups in a sample of patients with major depressive disorder: A preliminary study

Supplementary Methods

Participants

This study was approved by the Medical Research Ethics Committee of the Second Xiangya Hospital of Central South University, China. Sixty-four patients with first-episode drug-naive MDD (31 melancholic MDD and 33 non-melancholic MDD) were recruited from outpatients from May 4, 2014 to December 30, 2016, and 32 healthy controls were recruited from the community using printed advertisements. All participants were right-handed Han Chinese aged 18-45 years and with more than 9 years of education, and gave their written informed consents after fully understanding the study procedures. All patients were independently interviewed and diagnosed by two psychiatrists according to DSM-IV. The patients should be in their first major depressive episode with a total score of more than 17 in the 17-item Hamilton Rating Scale for Depression (HAM-D), and an illness duration of less than 12 months. The exclusion criteria were as follows: (1) concurrent axis I disorders, (2) history of any neurological disorders or substance abuse, and (3) exposure to psychotropic medications within at least 2 months. Healthy controls should have no first-degree family history of mental illness.

Image Acquisition and preprocessing

The resting-state functional images were acquired with echo planar imaging (EPI) sequence by the following parameters: repetition time/echo time (TR/TE) 2500/25 ms, 39 slices, 64 × 64 matrix, 90° flip angle, 24 cm field of view, 3.5 mm slice thickness, no gap, and 200 volumes lasting for 500s. All these imaging data were preprocessed using DPARSF (http://rfmri.org/DPARSF) in MATLAB[1]. To reduce effects of scanner signal stabilization, the first 10 images were discarded. Scans with maximum displacement of x, y, or z axis exceeding 2 mm or more than 2° of maximum angular rotation would be discarded. Functional and structural images were co-registered. Then structural images were normalized and segmented into gray, white and cerebrospinal fluid (CSF) partitions using the DARTEL technique. Several spurious covariates were removed from the realigned data, such as the signal from the ventricular seed-based ROI and the white matter-centered area, as well as the Friston 24-parameter model. The global signal was preserved[2]. After that, framewise displacement (FD) was calculated for all resting state volumes. All volumes with a FD greater than 0.5 mm were regressed out as nuisance covariates. Any scan with 50% of volumes removed was discarded. Then, functional time series were band pass filtered to select frequencies between 0.01-0.08 Hz and normalized by DARTEL into the Montreal Neurological Institute (MNI) template and then spatially smoothed at 6 mm full-width at half maximum (FWHM).

Meta-analyses for task-based fMRI studies

Study selection

A Embase, Pubmed and Web of Science database search was performed on all human studies between January 1, 2000 and May 1, 2022. The following terms and their derivatives were searched in [Title/Abstract]: *consummatory pleasure/consummatory anhedonia/reward outcome/reward gains/reward anticipation/anticipatory pleasure/anticipatory anhedonia/monetary incentive delay task/gambling task* and *major depressive*

disorder/depression/depressive disorder. To enhance search sensitivity, we also searched the reference lists manually as a supplement.

All available fMRI studies which investigated reward processing dysfunction in depressed individuals compared with healthy controls in all age groups were included. Specifically, studies with patients with MDD diagnosed by standardized diagnostic criteria were prioritized to be included in the meta-analysis. Studies with subjects with subthreshold depression and those who have first-degree relatives with a history of MDD were also included. We selected only studies that used instrumental reward tasks in which rewards were obtained by complete an action correctly rather presented passively (e.g., facial emotions or images)[3].

We excluded studies with depressed patients with other psychiatric disorders such as bipolar disorder and schizophrenia, or comorbid substance abuse disorders. Studies focused on reward-related decision-making or effort expenditure were also ruled out.

Data extraction and analysis

Data from the included studies such as demographic and methodological information, coordinates of significant brain areas, and statistics (e.g., t values or z values) were extracted. All coordinates were summarized in Montreal Neurological Institution (MNI) space. Studies reported outcomes in Talairach space were converted to MNI coordinates.

For studies which reported comparisons between depressed patients and healthy controls, SDM-PSI software version 6.22 (https://www.sdmproject.com/) was used. It combines several positive features from previous approaches, such as activation likelihood estimation and Multilevel Kernel Density Analysis, and introduced several improvements. Importantly, it provides a novel approach based on multiple imputation algorithms, which is able to reduce bias by including studies with NSUEs. Instead of simply excluding these studies (which would inflate result) or assuming them to have a null effect size (which could be too conservative), this method calculates maximum likelihood estimations of these NSUEs, creates realistic imputations, conducts a standard meta-analysis using restricted maximum-likelihood random-effect models for each set of imputations, and finally pools these meta-analyses. As recommend by SDM-PSI[4], the threshold was set at cluster-level $p < 0.05$ (threshold-free cluster enhancement-based family-wise error [FWE] correction) or $p < 0.001$ (uncorrected).

Given possible between-study heterogeneities introduced by age, sex, diagnoses and analysis method, our primary analyses were performed only in whole-brain studies with adult patients with acute MDD. Age, sex, education years and depressive severity were added as covariates in the comparative meta-analysis using the SDM-PSI. Specifically, the original scores of depressive severity in each study were standardized due to different rating scales used. We also included whole-brain studies with teenagers and patients with remitted MDD, subthreshold depression or high-risk of depression in the follow-up analyses to test whether outcomes would be significantly affected by these factors.

Supplementary Results

MDD patients revealed reduced activity in dorsal striatum during reward feedback

Overall, forty-three task-based fMRI studies examining reward prediction and experience met predefined the inclusion criteria, and 35 of them (590 patients with MDD, 147 subjects with remitted MDD or subthreshold depression or high-risk of depression, and 773 healthy controls)

were finally included in the meta-analyses (Figure S2, Table S3 and S4).
As shown in Figure 3 and Table S4, compared with healthy controls, patients with MDD exhibited increased activation in the supplementary motor area and decreased activation in the cerebellum lobule VI/fusiform gyrus ($p < 0.01$, uncorrected) during reward anticipation. During reward feedback, less blood-oxygen-level-dependent response in the putamen, caudate, and parahippocampal gyrus was observed in patients with MDD ($p < 0.001$, uncorrected). No results were reported when adopted a more conservative threshold FWE-corrected $p < 0.05$. We also performed a meta-analysis for all whole-brain studies regardless of age and diagnoses, results were similar except that no areas displayed increased activation in patients with MDD relative to healthy controls ($p < 0.01$, uncorrected).

Supplementary Discussion
The association of the dopaminergic system with the reward processing has been well-established in numerous preclinical and clinical studies. More recent research has expanded our understanding of reward mediation on a transmitter level, revealing interactions with other neurotransmitter systems like serotonergic, opioid, and glutamatergic systems[5]. This broader perspective suggests that distinct neurotransmitter systems may mediate various aspects of reward, with anticipatory pleasure being predominantly associated with dopaminergic mechanisms, while consummatory pleasure primarily linking to the opioid and serotonergic systems[5].
As for dopaminergic system, previous studies have demonstrated that rewarding experiences result in the release of dopamine in the striatum, particularly within the NAc, as evidenced by a reduction in the binding potential of dopamine receptor subtypes 2[6]. With regard to serotonergic system, studies have revealed that regions associated with reward processing receive substantial innervation from serotonergic neurons originating in the raphe nuclei. It has been reported that the serotonergic neurotransmitter system exerts a modulatory influence on the reward network through various receptors, including the 5-HT1A, 5-HT2A receptors, and the serotonin transporter (5-HTT)[7]. Evidence has indicated a significant reduction of 5-HTT in the midbrain and striatum in individuals with MDD[8]. It is important to note that while 5-HT signaling contributes to reward processing related to reward learning, anticipation, and outcome, the effects of SSRIs treatment on anhedonia are still unclear. Some studies reported reduced motivation and effort in depressed patients and enhanced effort expenditure in healthy subjects after an 8-week course of escitalopram treatment[9, 10]. This may be the reason why some patients did not benefit from Selective serotonin reuptake inhibitors (SSRIs) treatment. Similarly, inhibition of glutamate release also led to partial deficits in reward experiments[11]. It is suggested that the interplay between glutamate and striatal activity plays a pivotal role in modulating synaptic plasticity and neural excitability[12]. In particular, the influence of glutamatergic input from the frontal cortex has been underscored, with research revealing the impact of glutamatergic frontal neurons on dopaminergic neurotransmission in the ventral striatum through GABAergic neurons[13]. Moreover, there have been documented associations between glutamate levels and fronto-limbic functional connectivity[14]. As mentioned before, the opioid system in the NAc shell and the ventral pallidum plays a role in mediating consummatory pleasure. Opioid receptor antagonists have been suggested as a potential treatment for anhedonia based on robust preclinical data indicating the involvement of these receptors in modulating reward processing and stress regulation. Drawing from the available evidence, our hypothesis points that neurotransmitter systems, including

serotonergic, opioid, glutamatergic, and dopaminergic systems, exhibit varying degree of disruption within anhedonia subgroups of MDD. Patients exhibiting the most profound impairments in experiencing both anticipatory and consummatory pleasure are expected to demonstrate more severe dysfunction in neurotransmitter systems.

References:

(1) Chao-Gan, Y.; Yu-Feng, Z. DPARSF: A MATLAB Toolbox for "Pipeline" Data Analysis of Resting-State fMRI. *Front Syst Neurosci* **2010**, *4*, 13. DOI: 10.3389/fnsys.2010.00013.

(2) Hahamy, A.; Calhoun, V.; Pearlson, G.; Harel, M.; Stern, N.; Attar, F.; Malach, R.; Salomon, R. Save the global: global signal connectivity as a tool for studying clinical populations with functional magnetic resonance imaging. *Brain Connect* **2014**, *4* (6), 395-403. DOI: 10.1089/brain.2014.0244.

(3) Keren, H.; O'Callaghan, G.; Vidal-Ribas, P.; Buzzell, G. A.; Brotman, M. A.; Leibenluft, E.; Pan, P. M.; Meffert, L.; Kaiser, A.; Wolke, S.; et al. Reward Processing in Depression: A Conceptual and Meta-Analytic Review Across fMRI and EEG Studies. *Am J Psychiatry* **2018**, *175* (11), 1111-1120. DOI: 10.1176/appi.ajp.2018.17101124.

(4) Albajes-Eizagirre, A.; Solanes, A.; Radua, J. Meta-analysis of non-statistically significant unreported effects. *Stat Methods Med Res* **2019**, *28* (12), 3741-3754. DOI: 10.1177/0962280218811349.

(5) Hoflich, A.; Michenthaler, P.; Kasper, S.; Lanzenberger, R. Circuit Mechanisms of Reward, Anhedonia, and Depression. *Int J Neuropsychopharmacol* **2019**, *22* (2), 105-118. DOI: 10.1093/ijnp/pyy081 From NLM Medline.

(6) Jonasson, L. S.; Axelsson, J.; Riklund, K.; Braver, T. S.; Ogren, M.; Backman, L.; Nyberg, L. Dopamine release in nucleus accumbens during rewarded task switching measured by [(1)(1)C]raclopride. *Neuroimage* **2014**, *99*, 357-364. DOI: 10.1016/j.neuroimage.2014.05.047 From NLM Medline.

(7) Spies, M.; Knudsen, G. M.; Lanzenberger, R.; Kasper, S. The serotonin transporter in psychiatric disorders: insights from PET imaging. *Lancet Psychiatry* **2015**, *2* (8), 743-755. DOI: 10.1016/S2215-0366(15)00232-1 From NLM Medline.

(8) Gryglewski, G.; Lanzenberger, R.; Kranz, G. S.; Cumming, P. Meta-analysis of molecular imaging of serotonin transporters in major depression. *J Cereb Blood Flow Metab* **2014**, *34* (7), 1096-1103. DOI: 10.1038/jcbfm.2014.82 From NLM Medline.

(9) Pringle, A.; McCabe, C.; Cowen, P. J.; Harmer, C. J. Antidepressant treatment and emotional processing: can we dissociate the roles of serotonin and noradrenaline? *J Psychopharmacol* **2013**, *27* (8), 719-731. DOI: 10.1177/0269881112474523 From NLM Medline.

(10) Meyniel, F.; Goodwin, G. M.; Deakin, J. W.; Klinge, C.; MacFadyen, C.; Milligan, H.; Mullings, E.; Pessiglione, M.; Gaillard, R. A specific role for serotonin in overcoming effort cost. *Elife* **2016**, *5*. DOI: 10.7554/eLife.17282 From NLM Medline.

(11) Liu, Z.; Zhou, J.; Li, Y.; Hu, F.; Lu, Y.; Ma, M.; Feng, Q.; Zhang, J. E.; Wang, D.; Zeng, J.; et al. Dorsal raphe neurons signal reward through 5-HT and glutamate. *Neuron* **2014**, *81* (6), 1360-1374. DOI: 10.1016/j.neuron.2014.02.010 From NLM Medline.

(12) Gleich, T.; Lorenz, R. C.; Pohland, L.; Raufelder, D.; Deserno, L.; Beck, A.; Heinz, A.; Kuhn, S.; Gallinat, J. Frontal glutamate and reward processing in adolescence and adulthood. *Brain Struct Funct* **2015**, *220* (6), 3087-3099. DOI: 10.1007/s00429-014-0844-3 From NLM Medline.

(13) Carlsson, A.; Waters, N.; Carlsson, M. L. Neurotransmitter interactions in schizophrenia--therapeutic implications. *Biol Psychiatry* **1999**, *46* (10), 1388-1395. DOI: 10.1016/s0006-3223(99)00117-

1 From NLM Medline.
(14) Duncan, N. W.; Wiebking, C.; Tiret, B.; Marjanska, M.; Hayes, D. J.; Lyttleton, O.; Doyon, J.; Northoff, G. Glutamate concentration in the medial prefrontal cortex predicts resting-state cortical-subcortical functional connectivity in humans. *PLoS One* **2013**, *8* (4), e60312. DOI: 10.1371/journal.pone.0060312 From NLM Medline.
(15) Admon, R.; Nickerson, L. D.; Dillon, D. G.; Holmes, A. J.; Bogdan, R.; Kumar, P.; Dougherty, D. D.; Iosifescu, D. V.; Mischoulon, D.; Fava, M.; et al. Dissociable cortico-striatal connectivity abnormalities in major depression in response to monetary gains and penalties. *Psychol Med* **2015**, *45* (1), 121-131. DOI: 10.1017/s0033291714001123 From NLM.
(16) Admon, R.; Kaiser, R. H.; Dillon, D. G.; Beltzer, M.; Goer, F.; Olson, D. P.; Vitaliano, G.; Pizzagalli, D. A. Dopaminergic Enhancement of Striatal Response to Reward in Major Depression. *Am J Psychiatry* **2017**, *174* (4), 378-386. DOI: 10.1176/appi.ajp.2016.16010111 From NLM.
(17) Arrondo, G.; Segarra, N.; Metastasio, A.; Ziauddeen, H.; Spencer, J.; Reinders, N. R.; Dudas, R. B.; Robbins, T. W.; Fletcher, P. C.; Murray, G. K. Reduction in ventral striatal activity when anticipating a reward in depression and schizophrenia: a replicated cross-diagnostic finding. *Front Psychol* **2015**, *6*, 1280. DOI: 10.3389/fpsyg.2015.01280 From NLM.
(18) Chandrasekhar Pammi, V. S.; Pillai Geethabhavan Rajesh, P.; Kesavadas, C.; Rappai Mary, P.; Seema, S.; Radhakrishnan, A.; Sitaram, R. Neural loss aversion differences between depression patients and healthy individuals: A functional MRI investigation. *Neuroradiol J* **2015**, *28* (2), 97-105. DOI: 10.1177/1971400915576670.
(19) DelDonno, S. R.; Mickey, B. J.; Pruitt, P. J.; Stange, J. P.; Hsu, D. T.; Weldon, A. L.; Zubieta, J. K.; Langenecker, S. A. Influence of childhood adversity, approach motivation traits, and depression on individual differences in brain activation during reward anticipation. *Biol Psychol* **2019**, *146*, 107709. DOI: 10.1016/j.biopsycho.2019.05.009.
(20) Dillon, D. G.; Dobbins, I. G.; Pizzagalli, D. A. Weak reward source memory in depression reflects blunted activation of VTA/SN and parahippocampus. *Soc Cogn Affect Neurosci* **2014**, *9* (10), 1576-1583. DOI: 10.1093/scan/nst155.
(21) Dichter, G. S.; Kozink, R. V.; McClernon, F. J.; Smoski, M. J. Remitted major depression is characterized by reward network hyperactivation during reward anticipation and hypoactivation during reward outcomes. *J Affect Disord* **2012**, *136* (3), 1126-1134. DOI: 10.1016/j.jad.2011.09.048.
(22) Forbes, E. E.; Hariri, A. R.; Martin, S. L.; Silk, J. S.; Moyles, D. L.; Fisher, P. M.; Brown, S. M.; Ryan, N. D.; Birmaher, B.; Axelson, D. A.; et al. Altered striatal activation predicting real-world positive affect in adolescent major depressive disorder. *Am J Psychiatry* **2009**, *166* (1), 64-73. DOI: 10.1176/appi.ajp.2008.07081336.
(23) Gaillard, C.; Guillod, M.; Ernst, M.; Federspiel, A.; Schoebi, D.; Recabarren, R. E.; Ouyang, X.; Mueller-Pfeiffer, C.; Horsch, A.; Homan, P.; et al. Striatal reactivity to reward under threat-of-shock and working memory load in adults at increased familial risk for major depression: A preliminary study. *Neuroimage Clin* **2020**, *26*, 102193. DOI: 10.1016/j.nicl.2020.102193.
(24) Gorka, S. M.; Huggins, A. A.; Fitzgerald, D. A.; Nelson, B. D.; Phan, K. L.; Shankman, S. A. Neural response to reward anticipation in those with depression with and without panic disorder. *J Affect Disord* **2014**, *164*, 50-56. DOI: 10.1016/j.jad.2014.04.019.
(25) Gotlib, I. H.; Hamilton, J. P.; Cooney, R. E.; Singh, M. K.; Henry, M. L.; Joormann, J. Neural processing of reward and loss in girls at risk for major depression. *Arch Gen Psychiatry* **2010**, *67* (4), 380-387. DOI: 10.1001/archgenpsychiatry.2010.13.

(26) Gradin, V. B.; Kumar, P.; Waiter, G.; Ahearn, T.; Stickle, C.; Milders, M.; Reid, I.; Hall, J.; Steele, J. D. Expected value and prediction error abnormalities in depression and schizophrenia. *Brain* **2011**, *134* (Pt 6), 1751-1764. DOI: 10.1093/brain/awr059.
(27) Hägele, C.; Schlagenhauf, F.; Rapp, M.; Sterzer, P.; Beck, A.; Bermpohl, F.; Stoy, M.; Ströhle, A.; Wittchen, H. U.; Dolan, R. J.; et al. Dimensional psychiatry: reward dysfunction and depressive mood across psychiatric disorders. *Psychopharmacology (Berl)* **2015**, *232* (2), 331-341. DOI: 10.1007/s00213-014-3662-7 From NLM.
(28) Hall, G. B.; Milne, A. M.; Macqueen, G. M. An fMRI study of reward circuitry in patients with minimal or extensive history of major depression. *Eur Arch Psychiatry Clin Neurosci* **2014**, *264* (3), 187-198. DOI: 10.1007/s00406-013-0437-9.
(29) He, Z.; Zhang, D.; Muhlert, N.; Elliott, R. Neural substrates for anticipation and consumption of social and monetary incentives in depression. *Soc Cogn Affect Neurosci* **2019**, *14* (8), 815-826. DOI: 10.1093/scan/nsz061.
(30) Jin, J.; Narayanan, A.; Perlman, G.; Luking, K.; DeLorenzo, C.; Hajcak, G.; Klein, D. N.; Kotov, R.; Mohanty, A. Orbitofrontal cortex activity and connectivity predict future depression symptoms in adolescence. *Biol Psychiatry Cogn Neurosci Neuroimaging* **2017**, *2* (7), 610-618. DOI: 10.1016/j.bpsc.2017.02.002 From NLM.
(31) Johnston, B. A.; Tolomeo, S.; Gradin, V.; Christmas, D.; Matthews, K.; Steele, J. D. Failure of hippocampal deactivation during loss events in treatment-resistant depression. *Brain* **2015**, *138* (Pt 9), 2766-2776. DOI: 10.1093/brain/awv177.
(32) Knutson, B.; Bhanji, J. P.; Cooney, R. E.; Atlas, L. Y.; Gotlib, I. H. Neural responses to monetary incentives in major depression. *Biol Psychiatry* **2008**, *63* (7), 686-692. DOI: 10.1016/j.biopsych.2007.07.023 From NLM.
(33) Kumar, P.; Goer, F.; Murray, L.; Dillon, D. G.; Beltzer, M. L.; Cohen, A. L.; Brooks, N. H.; Pizzagalli, D. A. Impaired reward prediction error encoding and striatal-midbrain connectivity in depression. *Neuropsychopharmacology* **2018**, *43* (7), 1581-1588. DOI: 10.1038/s41386-018-0032-x.
(34) Liu, W. H.; Valton, V.; Wang, L. Z.; Zhu, Y. H.; Roiser, J. P. Association between habenula dysfunction and motivational symptoms in unmedicated major depressive disorder. *Soc Cogn Affect Neurosci* **2017**, *12* (9), 1520-1533. DOI: 10.1093/scan/nsx074.
(35) Luking, K. R.; Pagliaccio, D.; Luby, J. L.; Barch, D. M. Depression Risk Predicts Blunted Neural Responses to Gains and Enhanced Responses to Losses in Healthy Children. *J Am Acad Child Adolesc Psychiatry* **2016**, *55* (4), 328-337. DOI: 10.1016/j.jaac.2016.01.007.
(36) Mori, A.; Okamoto, Y.; Okada, G.; Takagaki, K.; Jinnin, R.; Takamura, M.; Kobayakawa, M.; Yamawaki, S. Behavioral activation can normalize neural hypoactivation in subthreshold depression during a monetary incentive delay task. *J Affect Disord* **2016**, *189*, 254-262. DOI: 10.1016/j.jad.2015.09.036 From NLM.
(37) Olino, T. M.; McMakin, D. L.; Dahl, R. E.; Ryan, N. D.; Silk, J. S.; Birmaher, B.; Axelson, D. A.; Forbes, E. E. "I won, but I'm not getting my hopes up": depression moderates the relationship of outcomes and reward anticipation. *Psychiatry Res* **2011**, *194* (3), 393-395. DOI: 10.1016/j.pscychresns.2011.04.009.
(38) Olino, T. M.; McMakin, D. L.; Morgan, J. K.; Silk, J. S.; Birmaher, B.; Axelson, D. A.; Williamson, D. E.; Dahl, R. E.; Ryan, N. D.; Forbes, E. E. Reduced reward anticipation in youth at high-risk for unipolar depression: a preliminary study. *Developmental cognitive neuroscience* **2014**, *8*, 55-64. DOI: 10.1016/j.dcn.2013.11.005.
(39) Pizzagalli, D. A.; Holmes, A. J.; Dillon, D. G.; Goetz, E. L.; Birk, J. L.; Bogdan, R.; Dougherty, D. D.;

Iosifescu, D. V.; Rauch, S. L.; Fava, M. Reduced caudate and nucleus accumbens response to rewards in unmedicated individuals with major depressive disorder. *Am J Psychiatry* **2009**, *166* (6), 702-710. DOI: 10.1176/appi.ajp.2008.08081201.

(40) Redlich, R.; Dohm, K.; Grotegerd, D.; Opel, N.; Zwitserlood, P.; Heindel, W.; Arolt, V.; Kugel, H.; Dannlowski, U. Reward Processing in Unipolar and Bipolar Depression: A Functional MRI Study. *Neuropsychopharmacology* **2015**, *40* (11), 2623-2631. DOI: 10.1038/npp.2015.110.

(41) Remijnse, P. L.; Nielen, M. M.; van Balkom, A. J.; Hendriks, G. J.; Hoogendijk, W. J.; Uylings, H. B.; Veltman, D. J. Differential frontal-striatal and paralimbic activity during reversal learning in major depressive disorder and obsessive-compulsive disorder. *Psychol Med* **2009**, *39* (9), 1503-1518. DOI: 10.1017/S0033291708005072.

(42) Robinson, O. J.; Cools, R.; Carlisi, C. O.; Sahakian, B. J.; Drevets, W. C. Ventral striatum response during reward and punishment reversal learning in unmedicated major depressive disorder. *Am J Psychiatry* **2012**, *169* (2), 152-159. DOI: 10.1176/appi.ajp.2011.11010137.

(43) Sankar, A.; Yttredahl, A. A.; Fourcade, E. W.; Mickey, B. J.; Love, T. M.; Langenecker, S. A.; Hsu, D. T. Dissociable Neural Responses to Monetary and Social Gain and Loss in Women With Major Depressive Disorder. *Front Behav Neurosci* **2019**, *13*, 149. DOI: 10.3389/fnbeh.2019.00149 From NLM.

(44) Satterthwaite, T. D.; Kable, J. W.; Vandekar, L.; Katchmar, N.; Bassett, D. S.; Baldassano, C. F.; Ruparel, K.; Elliott, M. A.; Sheline, Y. I.; Gur, R. C.; et al. Common and Dissociable Dysfunction of the Reward System in Bipolar and Unipolar Depression. *Neuropsychopharmacology* **2015**, *40* (9), 2258-2268. DOI: 10.1038/npp.2015.75.

(45) Schneider, M.; Elbau, I. G.; Nantawisarakul, T.; Pöhlchen, D.; Brückl, T.; Be, C. W. G.; Czisch, M.; Saemann, P. G.; Lee, M. D.; Binder, E. B.; et al. Pupil Dilation during Reward Anticipation Is Correlated to Depressive Symptom Load in Patients with Major Depressive Disorder. *Brain sciences* **2020**, *10* (12). DOI: 10.3390/brainsci10120906 From NLM.

(46) Segarra, N.; Metastasio, A.; Ziauddeen, H.; Spencer, J.; Reinders, N. R.; Dudas, R. B.; Arrondo, G.; Robbins, T. W.; Clark, L.; Fletcher, P. C.; et al. Abnormal Frontostriatal Activity During Unexpected Reward Receipt in Depression and Schizophrenia: Relationship to Anhedonia. *Neuropsychopharmacology* **2016**, *41* (8), 2001-2010. DOI: 10.1038/npp.2015.370.

(47) Sharp, C.; Kim, S.; Herman, L.; Pane, H.; Reuter, T.; Strathearn, L. Major depression in mothers predicts reduced ventral striatum activation in adolescent female offspring with and without depression. *J Abnorm Psychol* **2014**, *123* (2), 298-309. DOI: 10.1037/a0036191.

(48) Smoski, M. J.; Felder, J.; Bizzell, J.; Green, S. R.; Ernst, M.; Lynch, T. R.; Dichter, G. S. fMRI of alterations in reward selection, anticipation, and feedback in major depressive disorder. *J Affect Disord* **2009**, *118* (1-3), 69-78. DOI: 10.1016/j.jad.2009.01.034.

(49) Smoski, M. J.; Rittenberg, A.; Dichter, G. S. Major depressive disorder is characterized by greater reward network activation to monetary than pleasant image rewards. *Psychiatry Res* **2011**, *194* (3), 263-270. DOI: 10.1016/j.pscychresns.2011.06.012.

(50) Steele, J. D.; Kumar, P.; Ebmeier, K. P. Blunted response to feedback information in depressive illness. *Brain* **2007**, *130* (Pt 9), 2367-2374. DOI: 10.1093/brain/awm150.

(51) Stoy, M.; Schlagenhauf, F.; Sterzer, P.; Bermpohl, F.; Hägele, C.; Suchotzki, K.; Schmack, K.; Wrase, J.; Ricken, R.; Knutson, B.; et al. Hyporeactivity of ventral striatum towards incentive stimuli in unmedicated depressed patients normalizes after treatment with escitalopram. *J Psychopharmacol* **2012**, *26* (5), 677-688. DOI: 10.1177/0269881111416686 From NLM.

(52) Stringaris, A.; Vidal-Ribas Belil, P.; Artiges, E.; Lemaitre, H.; Gollier-Briant, F.; Wolke, S.; Vulser, H.;

Miranda, R.; Penttila, J.; Struve, M.; et al. The Brain's Response to Reward Anticipation and Depression in Adolescence: Dimensionality, Specificity, and Longitudinal Predictions in a Community-Based Sample. *Am J Psychiatry* **2015**, *172* (12), 1215-1223. DOI: 10.1176/appi.ajp.2015.14101298.

(53) Simmons, W. K.; Burrows, K.; Avery, J. A.; Kerr, K. L.; Bodurka, J.; Savage, C. R.; Drevets, W. C. Depression-Related Increases and Decreases in Appetite: Dissociable Patterns of Aberrant Activity in Reward and Interoceptive Neurocircuitry. *Am J Psychiatry* **2016**, *173* (4), 418-428. DOI: 10.1176/appi.ajp.2015.15020162.

(54) Takamura, M.; Okamoto, Y.; Okada, G.; Toki, S.; Yamamoto, T.; Ichikawa, N.; Mori, A.; Minagawa, H.; Takaishi, Y.; Fujii, Y.; et al. Patients with major depressive disorder exhibit reduced reward size coding in the striatum. *Prog Neuropsychopharmacol Biol Psychiatry* **2017**, *79* (Pt B), 317-323. DOI: 10.1016/j.pnpbp.2017.07.006 From NLM.

(55) Ubl, B.; Kuehner, C.; Kirsch, P.; Ruttorf, M.; Diener, C.; Flor, H. Altered neural reward and loss processing and prediction error signalling in depression. *Soc Cogn Affect Neurosci* **2015**, *10* (8), 1102-1112. DOI: 10.1093/scan/nsu158 From NLM.

(56) Ubl, B.; Kuehner, C.; Kirsch, P.; Ruttorf, M.; Flor, H.; Diener, C. Neural reward processing in individuals remitted from major depression. *Psychol Med* **2015**, *45* (16), 3549-3558. DOI: 10.1017/s0033291715001452 From NLM.

(57) Wiggins, J. L.; Schwartz, K. T.; Kryza-Lacombe, M.; Spechler, P. A.; Blankenship, S. L.; Dougherty, L. R. Neural reactivity to reward in school-age offspring of depressed mothers. *J Affect Disord* **2017**, *214*, 81-88. DOI: 10.1016/j.jad.2017.03.020 From NLM.

Table S1 Demographic information of all included task-based fMRI studies

Order	Study	Sample type	MDD/HR (n1)	Depression	HC (n2)	Mean age (SD)	Female (%)	Task	Medicated
1	Admon et al. 2015[15]	MDD (DSM-IV) vs. HC	26	28.53 (BDI)	29	42.6 (11.7)<br>37.7 (14)	50<br>41.4	MID (reward feedback)	No
2	Admon et al. 2017[16]	MDD (DSM-IV) vs. HC	46	27.3 (BDI)	43	27<br>25.9	80<br>76.7	MID (reward anticipation/feedback)	No
3	Arrondo et al. 2015[17]	MDD (DSM-IV) vs. HC	24	32 (BDI)	21	33.08 (9.15)<br>34.33 (10.11)	29<br>19	MID (reward anticipation/feedback)	Yes
4	Chandrasekhar et al. 2015[18]	MDD vs. HC	10	-	10	31.9 (7.5)<br>27.5 (2.4)	20<br>10	Decision-making task (reward anticipation)	-
5	DelDonno et al. 2019[19]	MDD (DSM-IV) vs. HC	23	18.56 (HAMD-17)	27	25.09 (3.32)<br>29.15 (9.00)	70<br>85	MID (reward anticipation)	No
6	Dillon et al. 2014[20]	MDD (DSM-IV) vs. HC	21	23.62 (BDI)	21	34.3 (12.1)<br>36.6 (13.3)	50<br>42.8	Memory task (reward feedback)	No
7	Dichter et al. 2012[21]	rMDD vs. HC	19	2.63 (BDI)	19	23.6 (4)<br>27.9 (6.3)	21<br>36.8	MID (reward anticipation/feedback)	No
8	Forbes et al. 2009[22]	MDD (K-SADS-PL) vs. HC	15	-	28	13.5 (2.1)<br>13.1 (2.6)	70<br>75	Card guessing (reward anticipation/feedback)	No
9	Gaillard et al. 2020[23]	HR vs. HC	16	6.8 (BDI)	16	24.3 (4.1)<br>24.1 (3.7)	75<br>75	Fribourg reward task (reward anticipation/feedback)	No
10	Gorka et al. 2014[24]	MDD (DSM-IV) vs. HC	9	26.3 (HAMD-17)	18	25.4 (7.7)<br>29.5 (1.1)	66.7<br>72.2	Passive slot machine (reward anticipation)	Yes
11	Gotib et al. 2010[25]	HR vs. HC	13	1.46 (CDI)	13	12.2 (1.7)<br>12.6 (1.4)	100<br>100	MID (reward anticipation/feedback)	No
12	Gradin et al. 2011[26]	MDD (DSM-IV) vs. HC	15	22.93 (BDI)	17	45.27 (12.32)<br>40.64 (11.87)	60<br>59	Instrumental reward learning task (reward anticipation/feedback)	-
13	Hagele et al.	MDD (DSM-IV/ICD-	24	24.3 (BDI)	54	40.1 (11.6)	29	MID (reward	No

	2015[27]	10) vs. HC				37.7 (11.1)<br>38.4	24<br>25	anticipation)	
14	Hall et al. 2014[28]	MDD-FTE (DSM-IV) vs. HC	14 (BDI)	26.29 (BDI)	14	26.36 (7.43)<br>28.43 (8.19)<br>27.40	35.7<br>42.8<br>39	Money game (reward feedback)	Yes
		MDD-MTE (DSM-IV) vs. HC	15 (BDI)	13.40	15	47.67 (9.5)<br>46.33 (11.4)	73<br>66.7		
15	He et al. 2019[29]	Subthreshold depression vs. HC	21	16 (BDI)	20	19.76 (1.92)<br>19.45 (1.57)	38<br>60	MID and SID (reward anticipation/feedback)	No
16	Jin et al. 2017[30]	HR vs. HC	49	-	180	All: 15.25 (0.58)	100<br>100	Monetary gambling task (reward feedback)	No
17	Johnston et al. 2015[31]	Treatment resistant depression (MINI PLUS) vs. HC	19	32.42 (BDI)	21	50.79 (10.6)<br>46.14 (13.97)	79<br>71	Modified Pessiglione reward task (reward feedback)	Yes
18	Knutson et al. 2008[32]	MDD (DSM) vs. HC	14	25.38 (BDI)	12	30.7 (8.8)<br>28.6 (4.2)	64<br>67	MID (reward anticipation/feedback)	No
19	Kumar et al. 2018[33]	MDD (DSM-IV) vs. HC	25	26.26 (BDI)	26	25.25 (5.46)<br>26.31 (7.96)	76<br>73	Reward leaning task (reward feedback)	No
20	Liu et al. 2017[34]	MDD (DSM-IV) vs. HC	21	24.05 (HAMD-24)	17	30.7 (8.9)<br>28.3 (5.2)	57<br>59	Probabilistic reward learning task (reward feedback)	No
21	Luking et al. 2016[35]	HR vs. HC	16	53.19 (CDI)	32	9.2 (1.0)<br>9.0 (1.1)	50<br>53	Card guessing (reward feedback)	No
22	Mori et al. 2016[36]	Subthreshold depression vs. HC	15	18.1 (BDI)	15	18.5 (0.6)<br>19.1 (0.7)	60<br>47	MID (reward anticipation)	No
23	Olino et al. 2011[37]	MDD vs. HC	10	-	16	All: 13.3 (2.4)	73	Card guessing (reward anticipation)	No
24	Olino et al. 2014[38]	HR vs. HC	14	-	12	15.8 (3.0)<br>15.5 (2.5)<br>15.7	78.6<br>66.7<br>73	Card guessing (reward anticipation/feedback)	No
25	Pizzagalli et al.	MDD (DSM-IV) vs.	30	27.48 (BDI)	31	43.1 (12.9)	42	MID (reward	No

	2009[39]	HC				38.8 (14.4)	50	anticipation/feedback)	
26	Redlich et al. 2015[40]	MDD (DSM-IV) vs. HC	33	27.88 (BDI)	34	38.5 (12.1)<br>38.6 (12.3)	51.5<br>47	Card guessing (reward feedback)	Yes
27	Remijnse et al. 2009[41]	MDD (DSM-IV) vs. HC	20	25.6 (BDI)	27	35<br>32	40<br>70	Reversal learning task (reward feedback)	Yes
28	Robinson et al. 2012[42]	MDD (DSM-IV) vs. HC	13	20 (HAMD-21)	14	36 (11)<br>31 (6)	38<br>43	Pavlovian reward-punishment prediction (reward feedback)	No
29	Sankar et al. 2019[43]	MDD (DSM-IV) vs. HC	20	14.88 (HAMD-17)	20	30.00 (10.84)<br>30.25 (10.99)	100<br>100	MID (reward anticipation)	Yes
30	Satterthwaite et al. 2015[44]	MDD (DSM-IV) vs. HC	25	25.16 (BDI)	37	38.8 (12.8)<br>39.5 (11.6)	44<br>49	MID (reward feedback)	Yes
31	Schneider et al. 2020[45]	MDD and subthreshold depression (DSM-IV) vs. HC	41	-	25	35.9 (13.4)<br>32.1 (10.3)	66<br>48	Reward anticipation task	No
32	Segarra et al. 2016[46]	MDD (DSM-IV) vs. HC	24	32.62 (BDI)	21	33.08 (9.15)<br>34.33 (10.11)	29<br>19	Simulated slot machine (reward feedback)	Yes
33	Sharp et al. 2014[47]	HR + MDD (DSM-IV) vs. HC	33 (19+14)	16.09 (BDI)	19	13.3 (1.8)<br>13.7 (1.8)	100<br>100	Card guessing (reward anticipation/feedback)	-
34	Smoski et al. 2009[48]	MDD (DSM-IV) vs. HC	14	-	15	34.8 (14.3)<br>30.8 (9.7)	50<br>60	Wheel of fortune (reward anticipation/feedback)	No
35	Smoski et al. 2011[49]	MDD (DSM-IV) vs. HC	9	16.7 (BDI)	13	34.4 (15.1)<br>26.2 (6.3)	-	MID (reward anticipation/feedback)	Yes
36	Steele et al. 2007[50]	MDD (DSM-IV) vs. HC	15	36.9 (BDI)	14	45.9 (10.7)<br>43.0 (13.3)	73<br>50	Gambling task (reward feedback)	Yes
37	Stoy et al. 2012[51]	MDD (DSM-IV) vs. HC	15	23.6 (BDI)	15	41.9 (12.2)<br>39.5 (11.9)	33<br>33	MID (reward anticipation)	Yes

38	Stringaris et al. 2015[52]	MDD (DSM-IV) vs. HC	22	-	123	14.4 (0.3)<br>14.4 (0.4)	86<br>73	MID (reward anticipation)	Yes
		Subthreshold depression vs. HC	101	-	123	14.5 (0.4)<br>14.4 (0.4)	65<br>73	MID (reward anticipation)	Yes
39	Simmons et al. 2017[53]	MDD (DSM-IV) vs. HC	32	23 (HAMD-17)	16	35.8<br>33.9	72<br>69	Food task (reward feedback)	No
40	Takamura et al. 2017[54]	MDD (DSM-IV) vs. HC	12	30.8 (BDI)	12	44.0 (13.2)<br>38.3 (8.46)	50<br>50	MID (reward anticipation)	Yes
41	Ubl et al. 2015a[55]	MDD (DSM-IV) vs. HC	30	25.50 (BDI)	28	46 (11.85)<br>43.96 (12.85)	53.3<br>53.6	Modified version of a paradigm by Kirsch (reward anticipation/feedback)	No
42	Ubl et al. 2015b[56]	rMDD (DSM-IV) vs. HC	23	5.08 (BDI)	23	41.17 (12.08)<br>42.74 (12.19)	69.6<br>60.9	Monetary reward paradigm (reward anticipation/feedback)	No
43	Wiggins et al. 2017[57]	HR vs. HC	27	-	19	7.44 (0.73)<br>7.64 (0.84)	52<br>74	MID (reward anticipation/feedback)	No

Abbreviations: BDI, Beck Depression Inventory; CDI, Child Depression Inventory; HAMD, Hamilton Depression Scale; HC, healthy control; HR, high-risk group (individuals who have first-degree relatives with a history of mental illness); K-SADS-PL, the Schedule for affective disorder and schizophrenia-present and lifetime version; MDD, major depressive disorder; MID, monetary incentive delay task; MINI PLUS, Mini-international psychiatric interview; rMDD, remitted MDD; SD, standard deviation; SID, social incentive delay task.

Table S2 Summary of methods and results of all included studies

Study	Whole-brain analysis	Software	Reward phase	Comparative results	Activation (HC)	Activation (depression)
Admon et al. 2015	Yes	FSL	Outcome (reward > neutral)	L_Caudate, R_Caudate	-	-
Admon et al. 2017	Yes	SPM	Anticipation/outcome (reward > penalty)	No results	-	-
Arrondo et al. 2015	Yes	FSL	Anticipation (reward > neutral)	R_NAc, Frontal pole, L_NAc	No label found, SFG, L_Thalamus, R_NAc	-
Chandrasekhar et al. 2015	Yes	SPM	Anticipation (reward > loss)	R_MTG	-	-
DelDonno et al. 2019	Yes	SPM	Anticipation (reward > neutral)	Declive, Cuneus, Precuneus	-	-
Dillon et al. 2014	Yes	SPM	Outcome (reward > neutral)	R_parahippocampal gyrus, VTA/SN	-	-
Dichter et al. 2012	Yes	FSL	Anticipation (reward > neutral)	R_Anterior cerebellum, L_Caudate, R_ACC, L_ACC, R_MFG, R_FOC, L_Fusiform gyrus, R_Fusiform gyrus, R_Anterior paracingulate gyrus, L_Anterior parahippocampal gyrus, L_SPL, R_Precuneus, R_SMC, L_Posterior supramarginal gyrus, R_Posterior supramarginal gyrus	-	-
			Outcome (reward > neutral)	R_Angular gyrus, L_Central opercular cortex, R_ Central	-	-

				opercular cortex, L_PCC, L_FOC, R_FOC, R_Frontal pole, L_Insular cortex, R_Intracalcarine cortex, L_Planum polare, L_Precentral gyrus, R_Lateral SOG, L_Supramarginal gyrus (anterior), R_Supramarginal gyrus (posterior), L_Fusiform gyrus, L_Posterior STG, R_Temporal pole, R_Thalamus, L_Thalamus		
Forbes et al. 2009	No	SPM	Anticipation (reward > baseline)	L_Caudate, R_DLPFC, L_DLPFC,	R_Caudate body	L_DLPFC, R_DLPFC, R_Medial frontal gyrus, L_Orbital gyrus, R_MFG, L_MFG
			Outcome (reward > baseline)	L_Caudate, L_Medial frontal gyrus, L_DLPFC, R_DLPFC	L_Caudate head	R_DLPFC, L_Medial frontal gyrus, L_DLPFC, R_MFG, L_SFG, R_SFG
Gaillard et al. 2020	Yes	Others	Anticipation (reward > baseline)	R_Cuneus	L_Fusiform, R_ITG, L_Lateral occipital gyrus, R_Lateral occipital gyrus, R_SPL	L_Lateral occipital gyrus, R_Lateral occipital gyrus, L_SPL
Gorka et al. 2014	Yes	SPM	Anticipation (reward > neutral)	R_dorsal ACC	L_NAc	-
Gotib et al. 2010	No	SPM	Anticipation (reward > neutral)	R_Insula, L_Putamen, L_Insula	-	-
			Outcome (reward > neutral)	R_ACC, L_PCC, L_MCC, L_Putamen, L_ACC,	-	-

Gradin et al. 2011	Yes	SPM	Anticipation	R_Anterior thalamic nucleus, R_Hippocampus, R_paterior parahippocampal gyrus	L_Amygdala-hippocampal complex, R_Amygdala-hippocampal complex, L_Posterior parahippocampal gyrus, R_Posterior parahippocampal gyrus	L_Amygdala-hippocampal complex, R_Amygdala-hippocampal complex, L_Posterior parahippocampal gyrus
			Outcome (reward prediction error)	L_Putamen, L_NAc, R_NAc, L_Caudate, R_Caudate, Midbrain, R_Hippocampus	L_Putamen, R_Putamen, L_NAc, R_NAc, L_Amygdala-hippocampal complex, R_Amygdala-hippocampal complex, L_Caudate, R_Caudate, R_insula, Midbrain	L_Amygdala, R_Amygdala, R_Hippocampus, R_Insula
Hagele et al. 2015	Yes	SPM	Anticipation (reward > neutral)	R_VS	-	-
Hall et al. 2014	Yes	Others	Outcome (reward > punishment)	R_vmPFC, L_vmPFC	-	-
			Outcome (large reward > small reward)	R_NAc, L_Caudate, R_ACC, L_ACC	-	-
			Outcome (reward > punishment)	R_vmPFC, L_vmPFC, R_ACC, L_ACC, R_NAc, L_NAc, R_Hippocampus, L_Hippocampus	-	-
			Outcome (large reward > small reward)	R_NAc, L_NAc, R_ACC, L_ACC	-	-
He et al. 2019	Yes	SPM	Anticipation (reward > baseline)	No significant results	R_Caudate, L_IOG	R_Caudate, L_Fusiform, L_IOG
			Outcome (reward >	No significant results	L_IOG, R_Lingual gyrus,	R_IOG, R_Calcarine sulcus,

			baseline)		R_IOG, R_Putamen, L_Fusiform, L_MOG, R_Fusiform	R_MOG, L_ Calcarine sulcus, L_MOG, L_IOG, R_Fusiform, L_Fusiform, R_ITG, R_Putamen
Jin et al. 2017	No	SPM	Outcome (reward > neutral)	No significant results	-	-
Johnston et al. 2015	Yes	SPM	Outcome (reward > neutral)	R_Insula, L_Insula, L_Subgenual ACC, L_NAc, R_PCC, L_PCC	NAc/subgenual ACC/PCC, Lateral cortical regions	Subgenual ACC, Insula/amygdala/hippocampus, DLPFC
Knutson et al. 2008	Yes	Others	Anticipation (reward > neutral)	L_SFG, L_ACC, L_Precentral gyrus, R_Postcentral gyrus	R_ACC, L_Caudate, R_Putamen, R_NAc, L_NAc, R_Caudate, L_Putamen, Thalamus	R_SFG, R_NAc, R_Caudate, R_Putamen, L_NAc, L_Caudate tail, R_IFG, ACC, L_Putamen, Medial frontal gyrus, R_Precentral gyrus, R_MFG, R_Thalamus, L_Thalamus, R_Hippocampus, R_Postcentral gyrus, R_IPL, L_IPL, R_SPL, L_Precuneus, R_Precuneus
			Outcome (reward > neutral)	R_mPFC, L_Insula, R_Putamen, L_Putamen, L_SFG, L_Postcentral gyrus, L_IPL	L_mPFC, L_Caudate head, R_Caudate head, L_Putamen, Cingulate, L_Amygdala, R_Precentral gyrus, R_Caudate tail, L_Caudate tail, L_Hippocampus, R_Hippocampus, L_Postcentral gyrus, L_IPL, R_IPL, L_Precuneus, R_SPL	mPFC, R_Caudate head, L_Caudate tail, R_Caudate tail, L_PCC
Kumar et al. 2018	Yes	SPM	Outcome (reward prediction error)	No significant results	R_Visual cortex, R_Calcarine cortex, L_SPL, R_Anterior insula, R_Putamen	L_Visual cortex, L_IFG, L_SPL, L_Anterior insula, PCC, R_Precentral gyrus

Liu et al. 2017	Yes	SPM	Outcome (reward > neutral)	No significant results	-	-
Luking et al. 2016	Yes	SPM	Outcome (reward > neutral)	R_Caudate, R_ventral putamen, L_Anterior insula, R_Anterior insula	-	-
			Outcome (reward > loss)	R_Medial globus pallidus, R_Parahippocampal gyrus	-	-
Mori et al. 2016	Yes	SPM	Anticipation (reward > neutral)	L_Angular gyrus, R_Angular gyrus, R_MFG, R_IPL	-	-
Olino et al. 2011	No	SPM	Anticipation (reward > baseline)	L_Caudate body, L_Caudate tail, R_Caudate body, R_Caudate head	-	-
Olino et al. 2014	Yes	SPM	Anticipation (reward > baseline)	Caudate	-	-
			Outcome (reward > baseline)	No significant results	-	-
Pizzagalli et al. 2009	Yes	FSL	Anticipation (reward > neutral)	L_Putamen, R_Occipitofrontal fasciculus, R_MOG, R_Parahippocampal gyrus, R_IFG, L_IFG, R_MFG, L_MFG, R_Subgenual ACC, R_STG, L_Occipitofrontal fasciculus, L_IPL, R_Lingual gyrus, R_Cerebellum	-	-

			Outcome (reward > neutral)	R_Caudate, L_Caudate, L_NAc, R_Insula, L_Insula, R_IFG, R_MFG, R_Medial frontal gyrus, L_Precentral gyrus, R_Rostral ACC, R_Dorsal ACC, L_PCC, R_MTG, L_Cerebellum, L_Fusiform	-	-
Redlich et al. 2015	Yes	SPM	Outcome (reward > neutral)	No significant results	-	-
Remijnse et al. 2009	Yes	SPM	Outcome (reward > neutral)	R_STG, L_Precentral gyrus, R_Occipital cortex, L_Putamen	-	-
Robinson et al. 2012	Yes	SPM	Outcome (unexpected reward)	R_Putamen, L_MCC, L_MOG	-	-
Sankar et al. 2019	No	FSL	Anticipation (reward >neutral)	No significant results	L_NAc, R_NAc	L_NAc, R_NAc, R_Anterior insula, L_Anterior insula
Satterthwaite et al. 2015	No	FSL	Outcome (reward > loss)	L_VS, L_Anterior insula, VTA, ACC, R_Thalamus	-	-
Schneider et al. 2020	No	SPM	Anticipation (reward > neutral)	No significant results	-	-
Segarra et al. 2016	Yes	FSL	Outcome (reward > neutral)	Medial frontal cortex, R_VS, L_Lingual gyrus, L_OFC, R_ITG and MTG	-	-
Sharp et al. 2014	Yes	Others	Anticipation (reward > baseline)	No significant results	-	-
			Outcome (reward > baseline)	R_MTG, R_IFG, R_VS, R_IFG, R_IPL, R_Supramarginal gyrus, R_Medial frontal gyrus, L_Cingulate gyrus	-	-

Smoski et al. 2009	Yes	FSL	Anticipation (reward > neutral)	L_Parietal operculum cortex, L_Caudate, L_ACC, R_PCC, L_PCC, L_IFG, R_MFG, L_MFG, L_Frontal pole, L_Hippocampus, L_Lingual gyrus, L_IOG, R_IOG, L_SOG, L_Fusiform, R_Postcentral gyrus, L_Precentral gyrus, R_Precentral gyrus, L_Precuneous, R_Precuneous, L_Subcallosal cortex, L_ITG, L_MTG, L_Temporal pole, R_Thalamus, L_Thalamus	-	-
			Outcome (reward > neutral)	R_Angular gyrus, R_Cunues, R_IFG, R_Fusiform, R_Precuneous, L_Temporal pole, R_Thalamus, L_Thalamus, L_Cunues, R_MFG, L_Lingual gyrus, R_SOG, L_SOG	-	-
Smoski et al. 2011	Yes	FSL	Anticipation (reward > neutral)	R_OFC, L_Hippocampus, R_Occipital pole, R_Subcallosal cortex	-	-

			Outcome (reward > neutral)	No significant results	-	-
Steele et al. 2007	No	SPM	Outcome (reward > loss)	R_VS, L_VS	L_VS, R_VS	No significant results
Stoy et al. 2012	Yes	SPM	Anticipation (reward > neutral)	No significant results	R_Lentiform, L_Lentiform, Thalamus, L_MOG, Cuneus, L_MTG	No significant results
Stringaris et al. 2015	No	SPM	Anticipation (reward > neutral) (MDD vs. HC)	R_Caudate head, R_Caudate, L_Caudate, R_Medial frontal gyrus, R_SFG, L_SFG, L_MFG	-	-
			Anticipation (reward > neutral) (subthreshold depression vs. HC)	L_Caudate head, L_Putamen, R_Caudate head, R_Caudate	-	-
Simmons et al. 2017	No	Others	Outcome (reward > baseline)	R_Anterior insula, L_Dorsal mid-insula, R_Ventral pallidum, R_VS, L_VS, L_vmPFC, R_OFC, R_ Dorsal mid-insula, L_Anterior insula, L_Caudal anterior insula, R_Putamen	R_Visual cortex, L_Visual cortex, L_Insula, L_OFC, R_Dorsal mid-insula, R_Amygdala, R_OFC	-
Takamura et al. 2017	No	SPM	Anticipation (reward > neutral)	No significant results	-	-
Ubl et al. 2015a	No	SPM	Anticipation (high reward > neutral)	R_NAc, R_OFC, L_Rostral ACC	(whole-brain analysis): R_calcarine sulcus, R_SOG, R_IOG, L_MOG, L_IOG, L_thalamus, R_thalamus, L_pallidum, L_postcentral cortex, L_precentral cortex,	(whole-brain analysis): L_IOG, L_MOG, R_SOG, R_MOG, R_IOG, L_precentral cortex, L_SMC, R_SMC, L_SFG, R_lingual gyrus, R_thalamus, R_cerebelum_6, L_thalamus, L_hippocampus, R_SFG,

	L_SMC, R_MCC, R_SMC, R_ACC, L_ACC, R_fusiform, R_MFG, L_cerebelum_6,<br>L_fusiform, R_precentral cortex, R_postcentral cortex, L_precuneus, L_IPL, L_superior temporal pole, L_inferior OFC, L_MFG, R_rolandic operculum, R_superior temporal pole, L_MTG, R_caudate nucleus, R_putamen, L_STG, R_precuneus, R_MFG, L_caudate nucleus, R_SFG, L_supramarginal gyrus, L_crus1 cerebelum, L_MCC<br>R_cerebelum_4_5,<br>R_angular gyrus, L_SFG, L_putamen,<br>L_hippocampus,<br>R_supramarginal gyrus, L_lobule paracentral cortex, L_insula, L_SPL, L_rolandic operculum<br>R_postcentral cortex<br>L_lingual gyrus<br>L_calcarine sulcus<br>R_inferior OFC<br>L_IPL	L_cerebelum_6, R_putamen, L_pallidum, R_cerebelum_3, R_parahippocampal cortex, L_parahippocampal cortex<br>R_pallidum, L_postcentral cortex, L_lingual gyrus

					R_inferior operculum frontal cortex	
			Outcome (high reward > neutral)	No significant results	L_MOG, R_MOG, L_insula, R_insula, R_caudate nucleus, R_ACC, R_precentral cortex, R_angular gyrus, R_calcarine sulcus, L_pallidum, L_precentral cortex, L_SMC, R_fusiform, R_MCC, L_inferior operculum frontal cortex	R_lingual gyrus, R_calcarine sulcus, R_MOG, R_insula, R_inferior OFC, L_insula, R_Hippocampus, R_ACC, R_pallidum, R_middle OFC, R_thalamus, L_caudate nucleus, Vermis_1_2, L_thalamus, L_precentral cortex, R_SFG
Ubl et al. 2015b	No	SPM	Anticipation (reward > neutral)	L_Hippocampus, R_Amygdala, R_SFG	-	-
			Outcome (reward > neutral)	No significant results	-	-
Wiggins et al. 2017	Yes	Others	Anticipation (reward > baseline)	No significant results	-	-
			Outcome (reward > baseline)	R_DLPFC, R_Parahippocampal gyrus	-	-

Abbreviations: ACC, anterior cingulate cortex; DE, depressed-appetite decrease subgroup; DLPFC, dorsolateral prefrontal cortex; FOC, frontal orbital cortex; IFG, inferior frontal gyrus; IN, depressed-appetite increased subgroup; IPL, inferior parietal lobe; ITG, inferior temporal gyrus; MCC, middle cingulate cortex; MFG, middle frontal gyrus; MOG, middle occipital gyrus; mPFC, medial prefrontal cortex; MTG, middle temporal gyrus; NAc, nucleus accumbens; OFC, orbitofrontal cortex; PCC, posterior cingulate gyrus; SFG, superior frontal gyrus; SMC, supplementary motor cortex; SOG, superior occipital gyrus; SPL, superior parietal lobule; STG, superior temporal gyrus; vmPFC, ventral medial prefrontal cortex; VS, ventral striatum; VTA/SN, ventral tegmental area/substantia nigra.

Table S3 Results of meta-analysis for comparative studies with whole-brain method (depressed subjects vs. healthy controls)

Brain area	Voxels	MNI coordinates			Z score	P value
		x	y	z		
Anticipation*						
R_Supplementary motor area	35	6	4	46	2.840	0.002
R_Cerebellum lobule VI/fusiform gyrus	157	32	-62	-22	-2.788	0.004
Feedback**						
R_Lentiform/putamen	174	22	4	-4	-4.329	< 0.001
L_Caudate	129	-10	2	16	-4.033	< 0.001
L_Caudate	53	-6	4	-6	-3.825	< 0.001
R_Caudate	46	12	12	4	-3.500	< 0.001
R_Parahippocampal gyrus	11	20	-20	-18	-3.317	< 0.001

*: Results $p < 0.01$, uncorrected, cluster size > 10 voxels.

**: Results $p < 0.001$, uncorrected, cluster size > 10 voxels.

Table S4. Significant ALFF results (anticipatory and consummatory pleasure-related) across groups (GRF corrected, voxel level < 0.01, cluster level < 0.05).

Contrast	Cluster location	Peak MNI			F/T value
		x	y	z	
Comparison among 4 groups	Right_PHipp	42	-45	-9	6.08
	Left_Hipp	-30	-18	-15	6.22
	Left_PHipp/Hipp	-33	-39	-6	5.78
	Right_Caudate	18	21	6	5.55
SubA vs. SubB	Left_PHipp/Hipp	-21	-42	-3	-3.11
	Left_Hipp/Putamen	-30	-18	-15	-4.14
SubA vs. SubC	Left_Hipp	-30	-18	-15	-3.26
	Left_PHipp/Hipp	-33	-42	-3	-3.15
SubB vs. SubC:	Right_PHipp	33	-30	-15	2.98
SubA vs. HC	No brain region above the threshold				
SubB vs. HC	Right_PHipp/Hipp	33	-30	-15	3.81
SubC vs. HC	Right_Caudate	18	21	3	3.35

Abbreviations: ACC, anterior cingulate cortex; Hipp, Hippocampus; PHipp, Parahippocampal gyrus; SubA, SubB, and SubC, SubgroupA, SubgroupB, and SubgroupC; HC, healthy control.

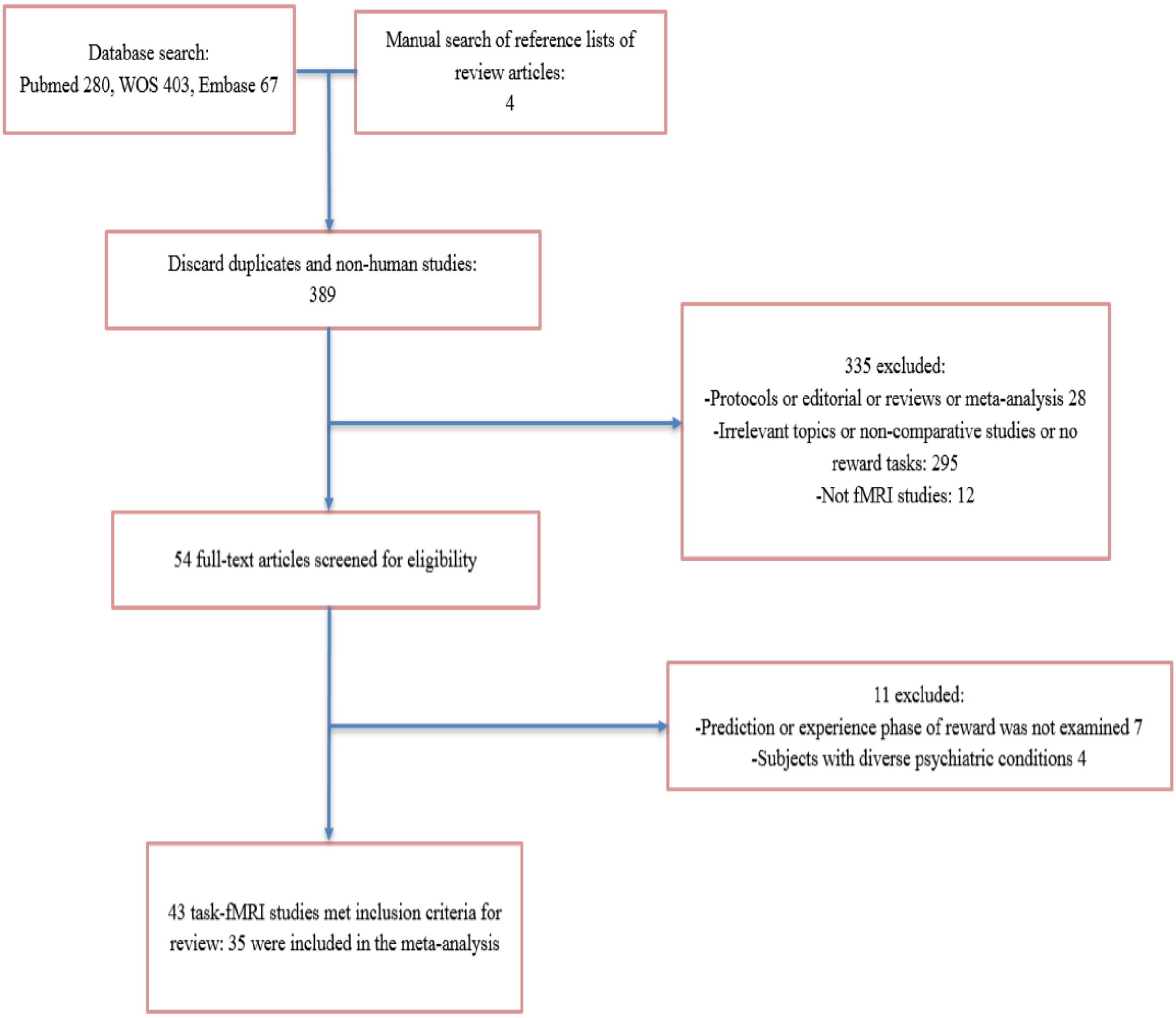

Figure S1 A flow diagram of study selection

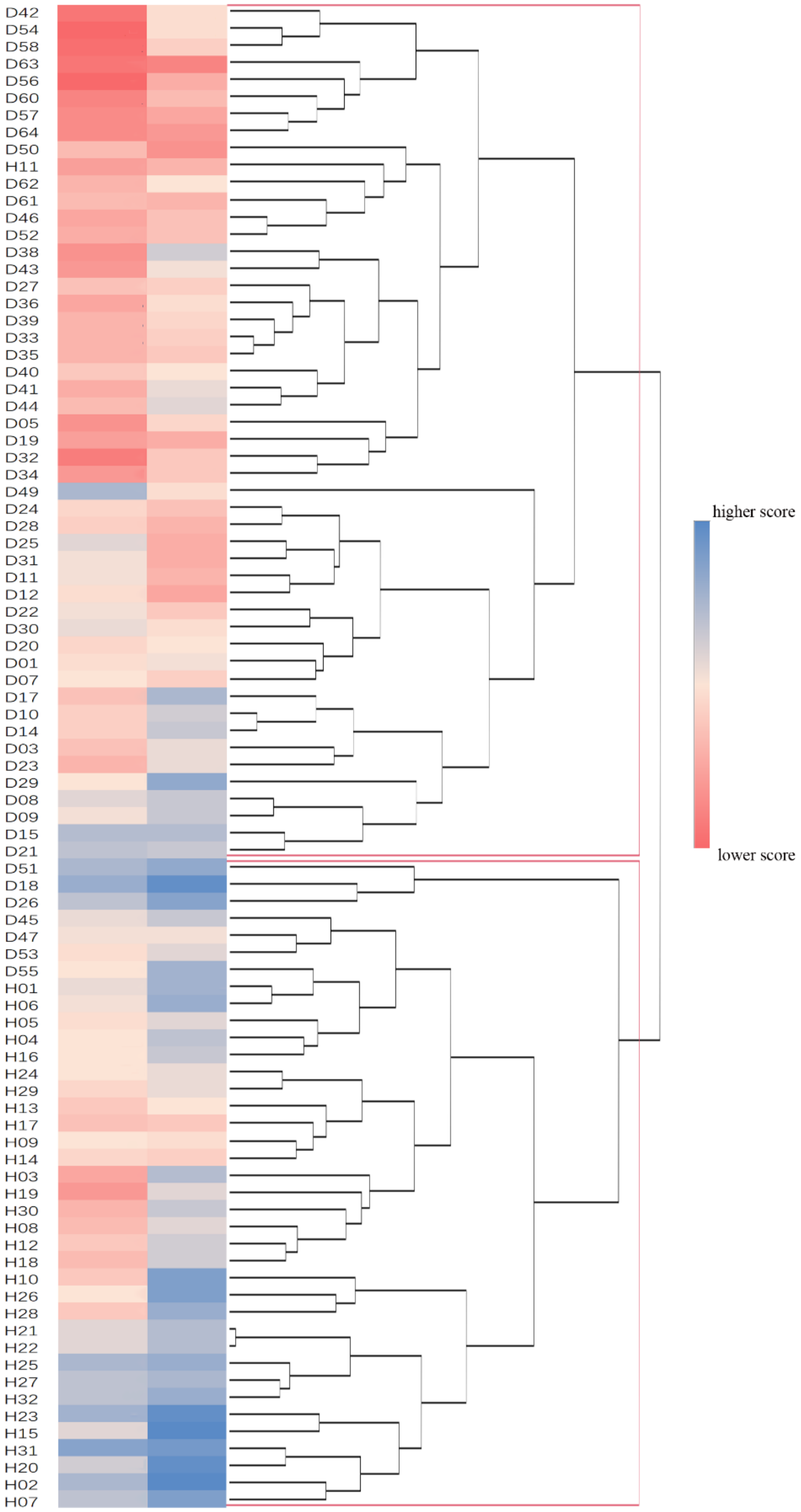

Figure S2 Classification of all participants to 2 clusters (the upper: group M, the lower: group N) based on scores of TEPS-anti and TEPS-con. The dendrogram showed the result of hierarchical clustering, and the heatmap demonstrated scores of TEPS-anti and TEPS-con of each individual. The left column indicated the ID of each individual (D is patient and H is healthy control).

Abbreviations: TEPS, the Temporal Experience of Pleasure Scale (TEPS-anti, anticipatory subscale of TEPS; TEPS-con, consummatory subscale of TEPS).

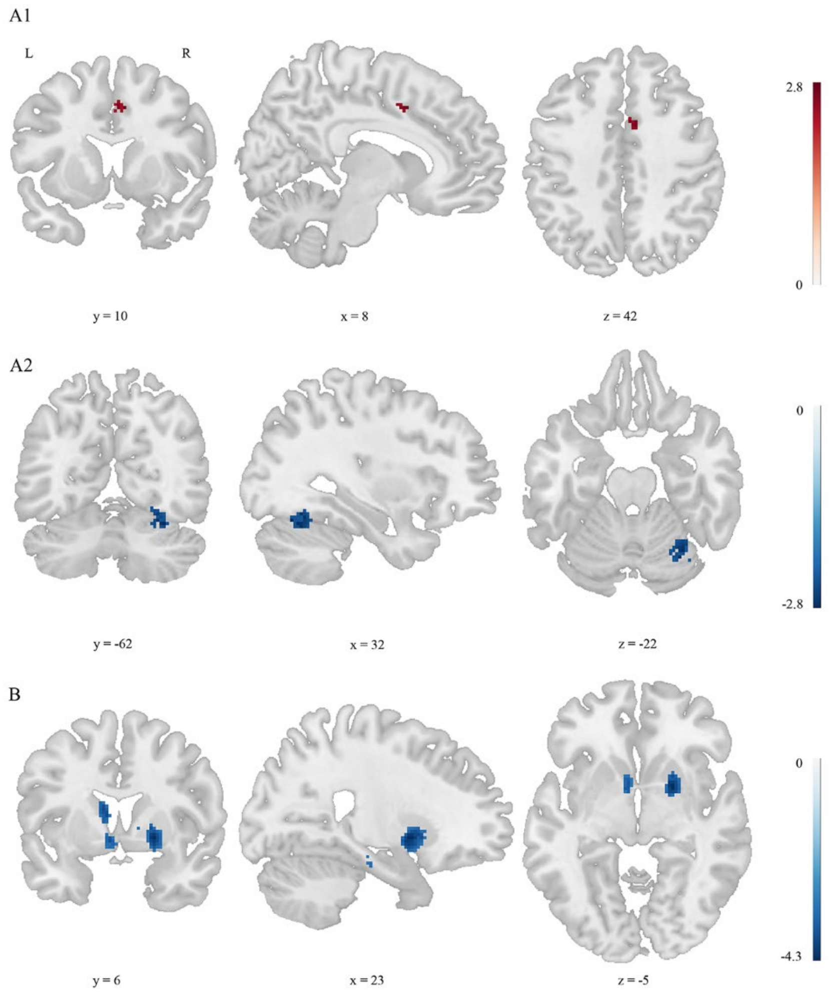

Figure S3 Differences of brain activation patterns in reward anticipation and feedback between patients with MDD and healthy controls

Image A depict brain regions in which patients with MDD exhibited greater activation (A1, right median cingulate gyrus) and lower activation (A2, cerebellum lobule VI/fusiform gyrus) than healthy controls during reward anticipation ($p < 0.01$, uncorrected); Image B shows brain regions in which MDD < healthy controls during reward feedback ($p < 0.001$, uncorrected). The colorbar indicates z scores.

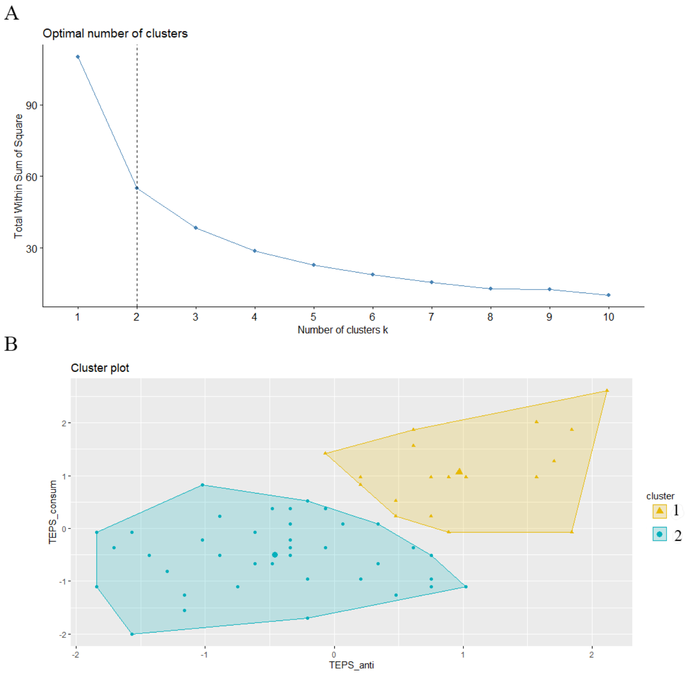

Figure S4 Elbow test for k-means analysis (A) and k-means clustering results (B) for all patients.

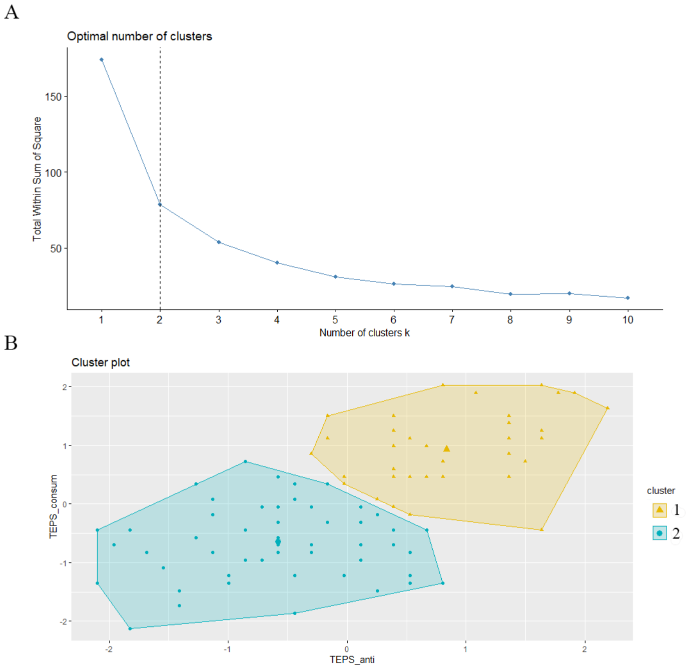

Figure S5 Elbow test for k-means analysis (A) and k-means clustering results (B) for all participants. Cluster 1 (higher TEPS scores) and Cluster 2 (lower TEPS scores) included 30 and 58 individuals, respectively. Specifically, 86% individuals in the Cluster 2 overlapped with those in the hierarchical clustering group M, and 61% individuals in the Cluster 1 were in the hierarchical clustering group N.
